# *In Silico* Genome-Wide Analysis of the Pear (*Pyrus bretschneideri*) *KNOX* Family and the Functional Characterization of *PbKNOX1*, an *Arabidopsis BREVIPEDICELLUS* Orthologue Gene, Involved in Cell Wall and Lignin Biosynthesis

**DOI:** 10.3389/fgene.2019.00632

**Published:** 2019-07-05

**Authors:** Xi Cheng, Manli Li, Muhammad Abdullah, Guohui Li, Jingyun Zhang, Muhammad Aamir Manzoor, Han Wang, Qing Jin, Taoshan Jiang, Yongping Cai, Dahui Li, Yi Lin

**Affiliations:** ^1^School of Life Science, Anhui Agricultural University, Hefei, China; ^2^Horticultural Institute, Anhui Academy of Agricultural Sciences, Hefei, China

**Keywords:** KNOX family, pear, stone cell, lignin, expression analysis, functional verification

## Abstract

Stone cells are a characteristic trait of pear fruit, but the contents and sizes of stone cells negatively correlate with fruit texture and flavor. Secondary cell wall thickening and lignification have been established as key steps of stone cell development. KNOTTED-LIKE HOMEOBOX (KNOX) proteins play important roles in plant cell growth and development, including cell wall formation and lignification. Although the characteristics and biological functions of KNOX proteins have been investigated in other plants, this gene family has not been functionally characterized in pear. Eighteen *PbKNOX* genes were identified in the present study, and all of the identified family members contained the KNOX I and/or KNOX II domains. Based on the phylogenetic tree and chromosomal localization, the 18 *PbKNOX* genes were divided into five subfamilies [*SHOOT MERISTEMLESS* (*STM*)-like, *BREVIPEDICELLUS* (*BP*)-like, *KNOTTED ARABIDOPSIS THALIANA 2/6* (*KNAT2/6*)-like, *KNAT7*-like, and *KNAT3-5*-like] and were distributed among 10 chromosomes. In addition, we identified 9, 11, and 11 *KNOX* genes in the genomes of grape, mei, and strawberry, respectively, and the greatest number of collinear *KNOX* gene pairs formed between pears and peaches. Analyses of the spatiotemporal expression patterns showed that the tissue specificity of *PbKNOX* gene expression was not very significant and that the level of the *PbKNOX1* transcript showed an opposite trend to the levels of stone cells and lignin accumulation. Furthermore, PbKNOX1 has high sequence identity and similarity with *Arabidopsis* BP. Compared with wild-type *Arabidopsis*, plants overexpressing *PbKNOX1* not only showed an approximately 19% decrease in the secondary cell wall thickness of vessel cells but also exhibited an approximately 13% reduction in the lignin content of inflorescence stems. Moreover, the expression of several genes involved in lignin biosynthesis was downregulated in transgenic lines. Based on our results, *PbKNOX1*/*BP* participates in cell wall-thickening and lignin biosynthesis and represses the transcription of key structural genes involved in lignin synthesis, providing genetic evidence for the roles of *KNOX* in cell wall thickening and lignin biosynthesis in pear.

## Introduction

*Pyrus bretschneideri* cv. “Dangshan Su” is a diploid species (2n = 34) of Chinese white pear (*P. bretschneideri* Rehd.) that originated from Dangshan County, Anhui Province, which is currently the largest cultivated area in China (Wu et al., 2013; Zhang et al., 2017). However, with the availability of a rich variety of fruits and improvements in the living standards of consumers, the inherent defects of a large size and the content of stone cell clusters in “Dangshan Su” have become increasingly prominent (Cheng et al., 2019). Stone cells are one of the key factors that determine the quality of pear fruits (Choi et al., 2007; Cai et al., 2010; Yan et al., 2014). The contents and sizes of stone cell clusters are significantly negatively correlated with the texture and sucrose content of pear fruit, and excessive stone cell clusters have resulted in a rough texture and poor flavor (Cheng et al., 2018; Xue et al., 2018). Therefore, the contents and sizes of stone cell clusters must be reduced to improve the quality of the “Dangshan Su” pear.

The stone cell of pear is a type of lignified cell, and the fully developed pear stone cell contains approximately 40% lignin (Tao et al., 2009; Brahem et al., 2017; Tian et al., 2017). During the process of stone cell formation, the thickening of the secondary cell wall (SCW) is accompanied by a large amount of lignin deposition. Stone cells belong to a family of SCW-forming cells (sclerenchyma cells) whose lumens are completely filled with highly lignified SCWs. The deposition of lignin on the cell wall and the thickening of SCWs have been determined to be key steps during the development of stone cells. Therefore, the identification of genes related to lignin metabolism and SCW development in pears is of great significance for the future regulation of stone cell formation. In recent years, there have been many reports on the identification of key structural genes in the lignin synthesis pathway, such as *HCT*, *COMT*, *CCoAOMT*, *CAD*, *CCR*, and *DIR* (Cheng et al., 2016, Cheng et al., 2017, Ma et al., 2017; Cheng et al., 2018). However, transcription factors (TFs) related to lignin synthesis and SCW development in pears have rarely been reported.

KNOTTED-LIKE HOMEOBOX (KNOX) TFs contain homeodomain (HD) and play important roles in plant apical meristem development, hormone metabolism, lignin biosynthesis, and SCW development (Zhao and Dixon, 2011; Townsley et al., 2013; Frangedakis et al., 2017). *KNOX* genes are primarily found in plant genomes in the form of a gene family. According to *KNOX* family studies in *Arabidopsis thaliana* [also known as *KNOTTED A. THALIANA* (*KNAT*)], the members of the *KNOX* gene family can be divided into two classes: class I (KNOX I) includes *KNAT1*/*BP*, *KNAT2*, *KNAT6*, and *STM*; class II (KNOX II) includes *KNAT3*, *KNAT4*, *KNAT5,* and *KNAT7* (Furumizu et al., 2015; Gao et al., 2015). 

Recently, *KNOX* family members have been used as transcriptional repressors to negatively regulate plant lignin metabolism pathways and SCW development (Wuddineh et al., 2016). For example, genes belonging to the KNOX I class have been isolated from *Arabidopsis* (*AtBP*), peach (*PpKNOPE1*), *Oryza sativa* (*OSH15*), *Panicum virgatum* (*PvKN1*), *Zea mays* (*ZmKN1*), and *Populus* (*ARBORKNOX2*; *ARK2*). Among these genes, *PpKNOPE1*, *ZmKN1*, *OSH15*, *ARK2*, and *PvKN1* are all orthologues of *AtBP* (Mele et al., 2003; Du et al., 2009; Testone et al., 2012; Townsley et al., 2013; Wuddineh et al., 2016; Yoon et al., 2017). Studies on *AtBP* and its orthologues have found that not only can they downregulate the expression of multiple structural genes (such as *PAL*, *CAD*, *CCR*, or *CesA*) in the lignin or cellulose synthesis pathway but they can also interact with the promoter regions of some lignin synthesis-related genes (*CAD*, *COMT*, or *CCoAOMT*) (Mele et al., 2003; Du et al., 2009; Testone et al., 2012). In addition, *AtKNAT7* and *PoptrKNAT7*, in the KNOX II class, have also been shown to negatively regulate lignin synthesis and SCW formation (Li et al., 2012). In summary, KNOX TFs can inhibit lignin biosynthesis, resulting in decreased lignin content and impaired SCW development in plants, making them ideal target genes for regulating the formation of stone cells in pear fruit.

Although the identification and functions of *KNOX* family members have been studied in *Arabidopsis*, poplar, peach, and switchgrass, the *KNOX* genes in pear remain unstudied. Researchers have not clearly determined which *KNOX* family members regulate lignin synthesis and SCW development in pears. The *KNOX* family was identified in pear at the whole genome level to address this problem. The molecular characteristics, gene structures, conserved domains, evolutionary relationships, duplication events, interspecies collinearity, and *cis*-acting elements of the *PbKNOX* family were systematically studied. Thus, combined with spatial-temporal expression pattern analyses and multiple sequence alignments, a putative ortholog of *AtBP*, named as *PbKNOX1*, was identified in the *PbKNOX* family. Finally, we investigated the functional characteristics of *PbKNOX1*. The results of this study not only provide a platform for the study of the *KNOX* family in pears but also provide new insights into the functions of *KNOX* in lignin metabolism and SCW formation. This study also lays a foundation for investigation of molecular regulation of pear lignin synthesis and improving fruit quality. 

## Materials and Methods

### Plant Materials and Treatment

The pear fruits were collected from pear trees planted in horticultural farms in Dangshan County, Anhui Province, on different days after flowering (DAF); the plants were cultivated under consistent water and fertilizer management conditions. We collected the pear fruits at eight developmental stages [including 15 DAF, 39 DAF, 47 DAF, 55 DAF, 63 DAF, 79 DAF, 102 DAF, and 145 DAF (mature period)] according to previous studies (Cheng et al., 2018, Cheng et al., 2019). The buds, annual stems, mature leaves, and flowers of the “Dangshan Su” pear were collected from the same pear tree. All the materials were stored in an ultra-low temperature freezer (−80°C) until subsequent experiments. 

The lignin and stone cell contents in pear fruit were determined using the methods described by Cai et al. (2010). The lignin content was reported as a percentage (calculated lignin content/calculated dry weight of flesh × 100%) (Cheng et al., 2019). Stone cell content = dry weight of stone cells/fresh weight of flesh × 100%.

Wild-type *A. thaliana* (Columbia-0) seeds were obtained from Nottingham Arabidopsis Stock Center (http://arabidopsis.info/BasicForm).

### Identification and Sequence Analysis of *PbKNOX* Family Members

*P. bretschneideri* (Chinese white pear) and *Prunus mume* (mei) genome files were downloaded from the GigaDB data (http://gigadb.org/dataset/100083). The genome databases of *Vitis vinifera* (grape) and *Fragaria vesca* (strawberry) were obtained from Phytozome database (https://phytozome.jgi.doe.gov/pz/portal.html).

Pear genomic data “Pyrus_bretschneideri_Chr_gene.pep_V121010” were downloaded from a website (http://gigadb.org/dataset/100083), and a local protein database was established using BioEdit software. Nine *Arabidopsis* KNOXs (Furumizu et al., 2015; Gao et al., 2015) were used as query sequences for searching against the local protein database to obtain candidate KNOX sequences in pear using BLASTp program (E-value = 0.001). After removing the repeated and redundant sequences, the conserved KNOX1 (PF03790) or KNOX2 (PF03791) domains in the candidate sequences of the *KNOX* family were detected using SMART (http://smart.embl-heidelberg.de/) and Pfam (http://pfam.xfam.org/). The sequences containing KNOX domain were considered as members of *KNOX* gene family. The isoelectric point (pI), number of amino acids (aa), and molecular weight (MW) of each KNOX protein was predicted with ProtParam3 (http://web.expasy.org/protparam/).

### Construction of the Phylogenetic Tree and Analysis of Gene Structures

All phylogenetic trees were constructed using MEGA 5.1 software. The sequence accession numbers used to construct the phylogenetic tree are listed in **Table S1**. Analysis of the structures of the *KNOX* genes was completed using the Gene Structure Display Server (http://gsds.cbi.pku.edu.cn/).

### Analyses of the Chromosome Locations and Gene Duplications of the *PbKNOX* Family

The information obtained from the genome database was used to determine the location of the *KNOX* family members on each chromosome. MapInspect software was used to visualize the distribution of *KNOX* genes on chromosomes (Lin et al., 2011). 

The determination of tandem or segmental duplications was performed using the methods reported by Cheng et al. (2017) and Zhang et al. (2014). The Ka (nonsynonymous substitution rate) and Ks (synonymous substitution rate) of duplicated gene pairs were calculated with DnaSP v5.0 software (Rozas and Rozas, 1995). Plant Genome Duplication Database (PGDD) (Lee et al., 2013) and Circos software (http://circos.ca/software/download/circos/) were used for the collinearity analysis. 

### Identification of Conserved Motifs

Conserved motifs were confirmed using Multiple EM for Motif Elicitation (MEME, http://meme-suite.org/). The specific parameters were a motif width greater than six and less than 200. 20 motifs were identified (Abdullah et al., 2018). 

### Analysis of the Type and Number of *cis*-Acting Regulatory Elements in *PbKNOX* Family Members

We obtained a promoter region of 1,500 bp upstream of the start codon of each *KNOX* CDS from the pear genome database to analyze the *cis*-acting regulatory elements in the *KNOX* promoters. Subsequently, the promoter sequences of *KNOX* genes were submitted to the PLANTCARE database (http://bioinformatics.psb.ugent.be/webtools/plantcare/html/) to identify the type and number of putative *cis*-acting regulatory elements.

### RNA Extraction, Reverse Transcription, and Quantitative Real-Time PCR Analysis

The total RNA was extracted from different pear tissues according to the instruction manual of the RNAprep Pure Plant Kit (Tiangen, China) and was reverse transcribed into cDNAs according to the instructions of the PrimeScript™ RT Reagent Kit (Perfect Real Time) (Takara, China). The TransStart Green qPCR SuperMix was purchased from TransGen Biotech, China. The same method was used for the isolation of RNA from *Arabidopsis*.

The pear *Tubulin* (GenBank accession no. AB239680.1) was used as reference (Cheng et al., 2018). Three biological replicates were performed for each sample. The relative expression levels of the genes were calculated according to 2^-ΔΔC^_T_ method (Livak and Schmittgen, 2001). Quantitative real-time PCR (qRT-PCR) was carried out using CFX96 instrument (Bio-Rad). The expression level of each *PbKNOX* was displayed in the form of a heatmap using TBtools software (https://github.com/CJ-Chen/TBtools/releases). The primers of qRT-PCR for each gene are listed in **Table S2**.

### Construction of the Eukaryotic Expression Vector and Genetic Transformation

Based on the sequence information from the pear genome, specific primers that were designed to amplify the CDS of *PbKNOX1* and restriction sites (*Bgl* II and *Spe* I) were added at both ends. The double-digested fragment was ligated with the expression vector pCambia1304 (GenBank: AF234300.1) using T_4_ DNA ligase (Takara, China) and verified by sequencing to obtain the plant expression vector pCambia1304-*PbKNOX1*. The vector was electroporated into *Agrobacterium tumefaciens* EHA105. 

After *A. tumefaciens*-mediated genetic transformation (floral dip method) of *Arabidopsis* (Clough and Bent, 1998), the harvested seeds were planted on Murashige and Skoog (MS) medium (containing 50 mg/L hygromycin) for the selection of transgene-positive plants. After the selection of positive plants, the young leaves were subjected to β-glucuronidase (GUS) staining to determine whether the exogenous gene was expressed in the plant. GUS expression was examined using a GUS histochemical assay kit (Real-Times, China) according to the manufacturer’s protocol.

### Histochemical Staining and Transmission Electron Microscopy

The inflorescence stems from 6 weeks old *Arabidopsis* plants (T_3_ generation) were hand sectioned. The sections from the bottom portion (approximately 4 cm in length) of the inflorescence stems were then placed on glass slides. After staining with the 2% phloroglucinol-HCl (Wiesner staining) and 1% toluidine blue, the sections were directly observed with a microscope (Pradhan Mitra and Loqué, 2014). 

The transmission electron microscopy (TEM) observations were performed using a previously described method (Anderson et al., 2015). The thickness of the cell walls was measured in the TEM images as previously described (Xue et al., 2018). Image-Pro Plus 6.0 software (Media Cybernetics, Inc., Rockville, MD, USA) was used to measure the cell wall thickness.

### Determination of the Lignin Content in *A. thaliana*

The lignin content of the inflorescence stem of *Arabidopsis* was determined using the acetyl bromide method (acetyl bromide-soluble lignin content), as described by Anderson et al. (2015). 

### Statistical Analyses

Statistical analyses were completed using the Statistical Program for Social Sciences (release 19.0, SPSS Inc., IBM, www.ibm.com) and Microsoft Excel 2010. 

## Results

### Identification and Characterization of Pear *KNOX* Family Members

We used the sequences of nine members of the *Arabidopsis KNOX* family as the query sequences and then identified 18 non-redundant *KNOX* family members in the pear genome, which were designated as *PbKNOX1*-*PbKNOX18* (**Table 1**). The protein properties, including the length of the aa sequence, MW, pI, and subcellular localization of the 18 *PbKNOX*-encoded proteins were analyzed using the ExPASy tool.

**Table 1:** Sequence characteristics of 18 *KNOX*s identified in pear.

Gene name	Gene code	Chr	Protein			
			Length (aa)	MW (kDa)	pI	Subcellular localization
PbKNOX1	Pbr019805.1	Chr15	397	44.9	6.03	Nucleus
PbKNOX2	Pbr029256.1	Chr8	399	45.3	6.06	Nucleus
PbKNOX3	Pbr003791.1	/	341	38.6	6.15	Nucleus
PbKNOX4	Pbr021763.1	Chr4	147	16.3	6.14	Nucleus
PbKNOX5	Pbr021766.1	Chr4	151	16.9	6.28	Cytoplasm
PbKNOX6	Pbr000101.2	Chr5	416	46.3	6.07	Nucleus
PbKNOX7	Pbr033829.1	Chr2	360	40.8	5.41	Nucleus
PbKNOX8	Pbr042526.1	Chr8	454	50. 6	6.07	Nucleus
PbKNOX9	Pbr005158.1	Chr9	333	37.6	6.32	Nucleus
PbKNOX10	Pbr031941.1	Chr10	391	43.8	6.32	Nucleus
PbKNOX11	Pbr016430.1	Chr12	320	35.9	5.09	Nucleus
PbKNOX12	Pbr001919.1	Chr14	197	21.1	4.74	Nucleus
PbKNOX13	Pbr007308.1	Chr2	359	40.7	5.48	Nucleus
PbKNOX14	Pbr027897.1	Chr15	454	50.7	6.21	Nucleus
PbKNOX15	Pbr034311.1	Chr15	358	40.5	5.29	Nucleus
PbKNOX16	Pbr001553.1	Chr6	159	17.5	6.01	Nucleus
PbKNOX17	Pbr033866.1	Chr2	434	48.7	5.32	Nucleus
PbKNOX18	Pbr029155.1	/	358	40.5	5.29	Nucleus

“/” indicates that the specific chromosome is not known.

As shown in **Table 1**, the lengths of the PbKNOX proteins ranged from 148 aa (PbKNOX4) to 455 aa (PbKNOX8 and PbKNOX14), and the molecular weights ranged from 16.3 kDa (PbKNOX4) to 50.7 kDa (PbKNOX14). Furthermore, the theoretical pI values of the *PbKNOX* family members were all less than 7, ranging from 5.09 (PbKNOX11) to 6.32 (PbKNOX9 and PbKNOX10). Analyses of the predicted subcellular localization revealed that most of the PbKNOX proteins were located in the nucleus, consistent with their functions as TFs.

### Evolutionary Analyses, Conserved Motif Divergence and Intron/Exon Organization of *PbKNOX* Genes

To further explore the characteristics of the *PbKNOX* family, we analyzed the gene structure and conserved motifs of each member of *PbKNOX* family, based on an intraspecific phylogenetic tree (**Figure S1**). As shown in **Figure S1A**, the *PbKNOX* family can be roughly divided into two subfamilies (class I and II), with *PbKNOX* class I containing eight members and *PbKNOX* class II containing 10 members, including *PbKNOX4*, *5*, *7*, *8*, *13*, *14*, *15*, *16*, *17*, and *18*. In the phylogenetic tree, the 12 *PbKNOX* genes formed six gene pairs (*PbKNOX7*/*PbKNOX17*, *PbKNOX15*/*PbKNOX18*, *PbKNOX8*/*PbKNOX14*, *PbKNOX5*/*PbKNOX16*, *PbKNOX6*/*PbKNOX10*, and *PbKNOX2*/*PbKNOX3*), most of which have higher bootstrap values (≥99).

The gene structures of the *PbKNOX* family members were compared using the online software Gene Structure Display Server (**Figure S1C**). All *PbKNOX* genes consist of exons and introns, and the number of introns ranges from 1 to 5. Among the *PbKNOX* family members, only *PbKNOX4* contains one intron. *PbKNOX5*, *PbKNOX12*, and *PbKNOX16* have two introns, while *PbKNOX3*, *PbKNOX9*, and *PbKNOX10* have three introns. Eight members of the *PbKNOX* family contain four introns. In addition, three members, *PbKNOX8*, *PbKNOX14*, and *PbKNOX17*, have five introns. In general, members with close kinship have similar gene structures, such as *PbKNOX15*/*PbKNOX18* and *PbKNOX5*/*PbKNOX16*.

**Figure 1:**
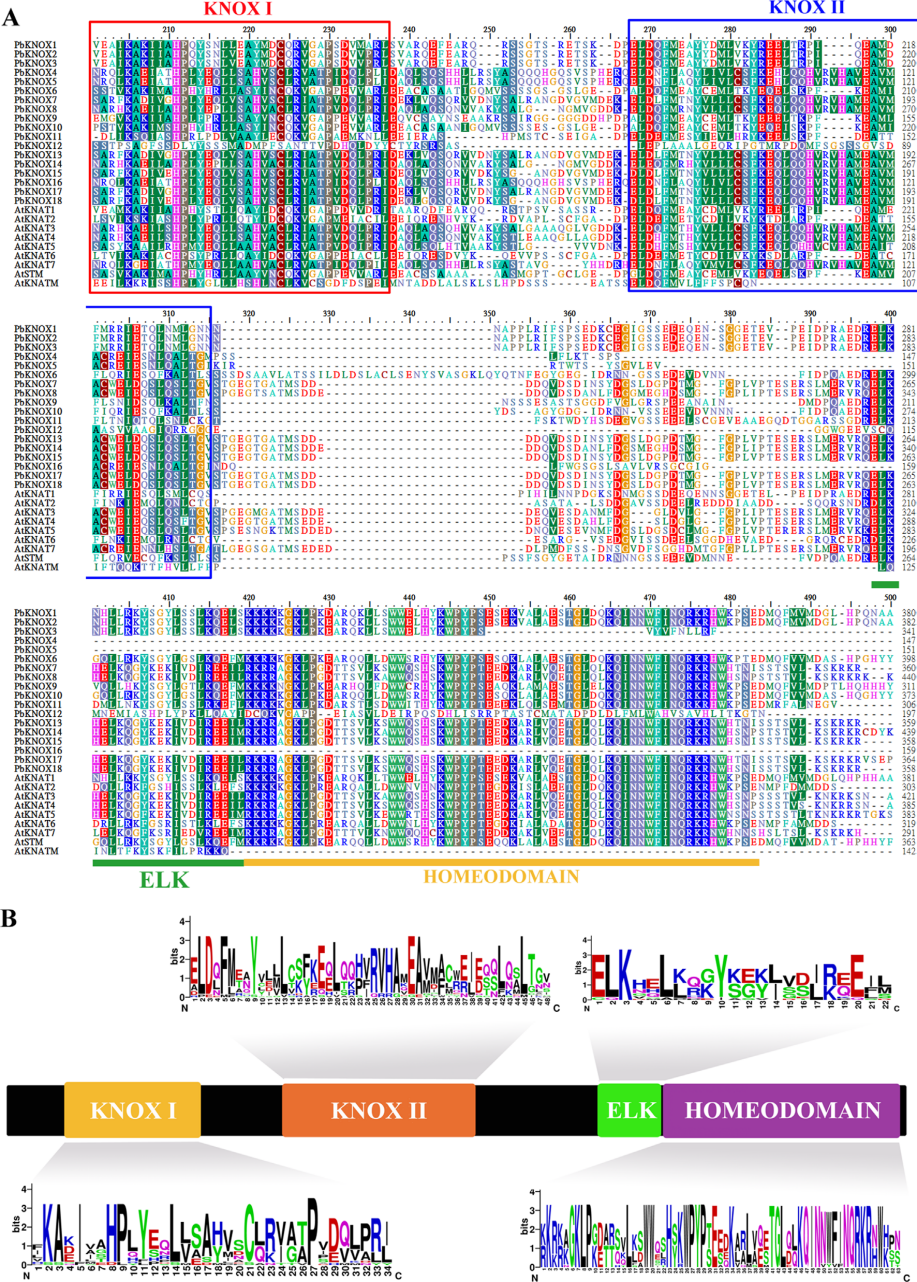
Multiple sequence alignment and domain compositions of PbKNOX proteins. **(A)** Multiple sequence alignment of KNOX proteins from various plants. **(B)** The distribution and composition of the characteristic motifs of PbKNOX proteins. The bit score represents the information content for each position in the sequence. The logo consists of stacks of symbols, one stack for each position in the sequence. The overall height of the stack indicates the sequence conservation at that position, while the height of symbols within the stack indicates the relative frequency of each amino acid at that position.

Subsequently, we identified 20 conserved motifs from the 18 PbKNOX proteins, using the MEME website, and annotated each motif using SMART and Pfam software (**Figure S1B**). The sequence composition and annotations of each motif are listed in **Table S3**. The results indicated that motif 1 contains ELK (PF03789) and HD (PF05920) domains, motif 2 encodes the KNOX I domain (PF03790), and motif 3, motif 6, and motif 8 together form the KNOX II domain (PF03791). In addition, all 18 PbKNOX proteins contain at least one of four domains (motif 2, motif 3, motif 6, and motif 8), further indicating that the results of the *PbKNOX* gene screening are reliable. Generally, PbKNOXs belonging to the same subfamily showed similar conserved motif types and distributions, which supports their close evolutionary relationships. Multiple sequence alignments of pear *KNOX* family members with *Arabidopsis* *KNOX* family members revealed that, although some members lacked conserved domains, most of the members contained four conserved domains (ELK, Homeobox_KN, KNOX I, and KNOX II). The order of distribution from the N-terminal to the C-terminal is as follows: KNOX I, KNOX II, ELK, and HD (**Figure 1A**). PbKNOX12 is a special member that lacks the KNOX2 and ELK domains and retains only the KNOX1 domain. Peach KNOPE2.1 presents a similar domain structure (**Figure S2**). In addition, we also analyzed the amino acid sequence composition of the four conserved domains of the pear *KNOX* family using the online website WebLogo (http://weblogo.berkeley.edu/logo.cgi) (**Figure 1B**).

### Chromosome Localization and Gene Duplication Analysis of *PbKNOX* Family Members

To clarify the distribution of *PbKNOX* genes on chromosomes and the mechanism of expansion within the whole genome, this study analyzed the chromosomal location and gene duplication events of *PbKNOX*s. Based on analyses of the physical chromosomal location, most *PbKNOX* genes were randomly distributed across 10 chromosomes (**Figure S3**); however, *PbKNOX3* and *PbKNOX18* were not located on any chromosome.

Among 18 *PbKNOX*s, we identified three genes pairs (*PbKNOX5/KNOX16*, *PbKNOX6/KNOX10*, and *PbKNOX8/KNOX14*), which displayed segmental duplication (**Figure S3**). In addition, the Ka/Ks ratio of duplication events was calculated by DnaSP v5.0 software (**Table S4**). The Ka/Ks ratios of the gene pairs displaying segmental duplication were less than 1, indicating that they might have undergone strong purification selection. 

### *In Silico* Genome-Wide and Interspecies Collinearity Analyses of the *KNOX*s in Different Rosids

**Figure 2:**
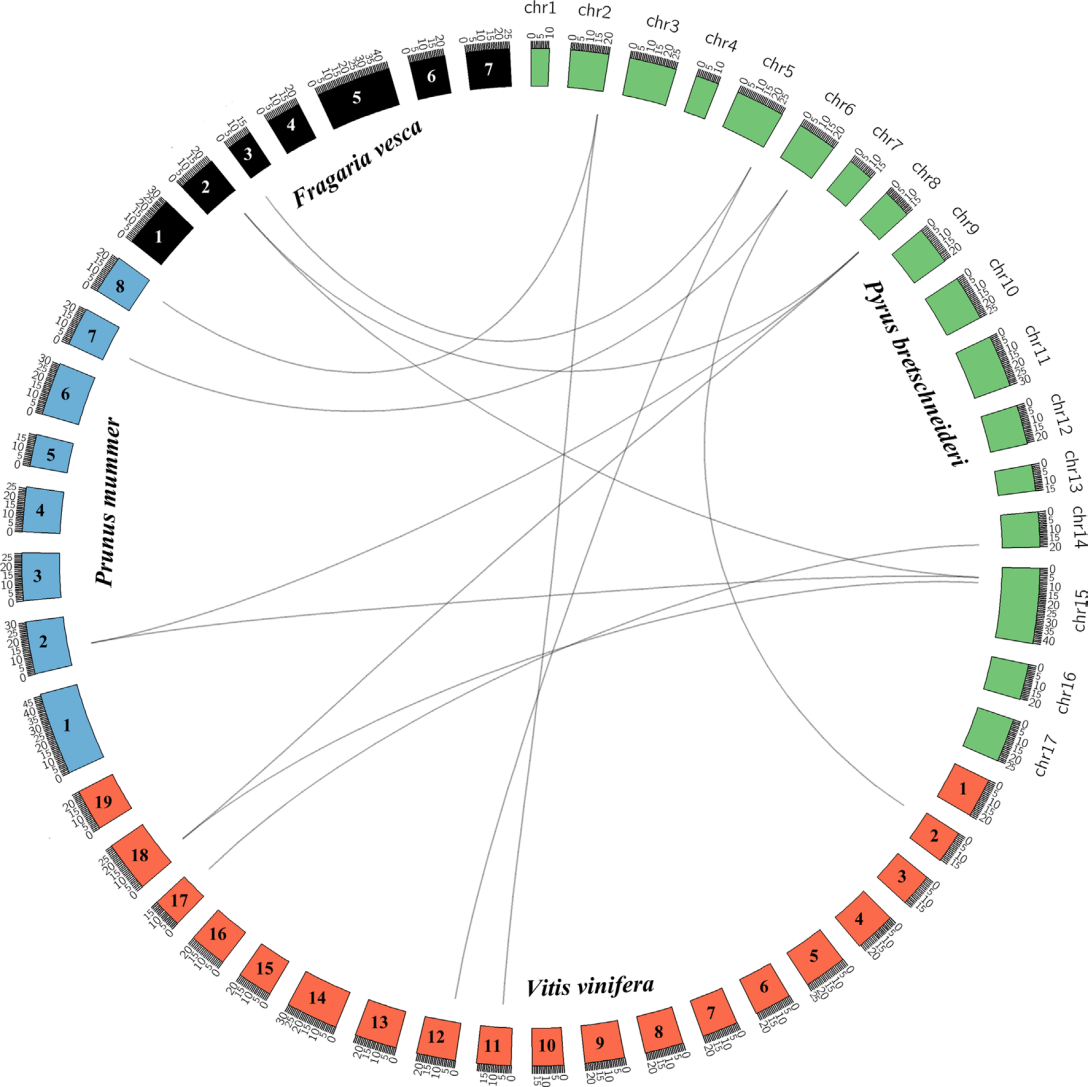
Interspecies collinearity analysis of KNOX regions across among *Pyrus bretschneideri* (pear) and *Prunus mume* (mei), *Vitis vinifera* (grape), and *Fragaria vesca* (strawberry).

Pear, mei, strawberry, and grape all originated from a common ancestor and belong to the rosid family (Reveal and Chase, 2011). We analyzed the collinearity of the *KNOX* genes in the genomes of these five rosids using the PGDD website and Circos software to elucidate the potential evolutionary mechanisms (**Figure 2**). We identified 9, 11, and 11 *KNOX*s from the genomes of grape, mei, and strawberry, respectively (**Table S5**). We constructed an interspecies phylogenetic tree of pear *KNOX* genes (*PbKNOX*), grape *KNOX* genes (*VvKNOX*), strawberry *KNOX* genes (*FvKNOX*), and mei *KNOX* genes (*PmKNOX*) and analyzed their conserved motifs and gene structures (**Figure S4**).

Subsequently, we identified 13 collinear gene pairs from four rosids, including three gene pairs between pear and strawberry, four between pear and mei, and six between pear and grape (**Figure S5** and **Table S6**). It is worth noting that some *PbKNOX*s have collinear relationships with the members of the *FvKNOX*, *PmKNOX*, and *VvKNOX* families, such as *PbKNOX1* (Pbr019805.1) and *PbKNOX2* (Pbr029256.1). These results indicated that these gene pairs likely appeared in the common ancestor before the divergence into pear, mei, strawberry, and grape. Interestingly, *PbKNOX*6 only has collinear relationships with *VvKNOX* and *FvKNOX* and not with *PmKNOX*.

**Figure 3:**
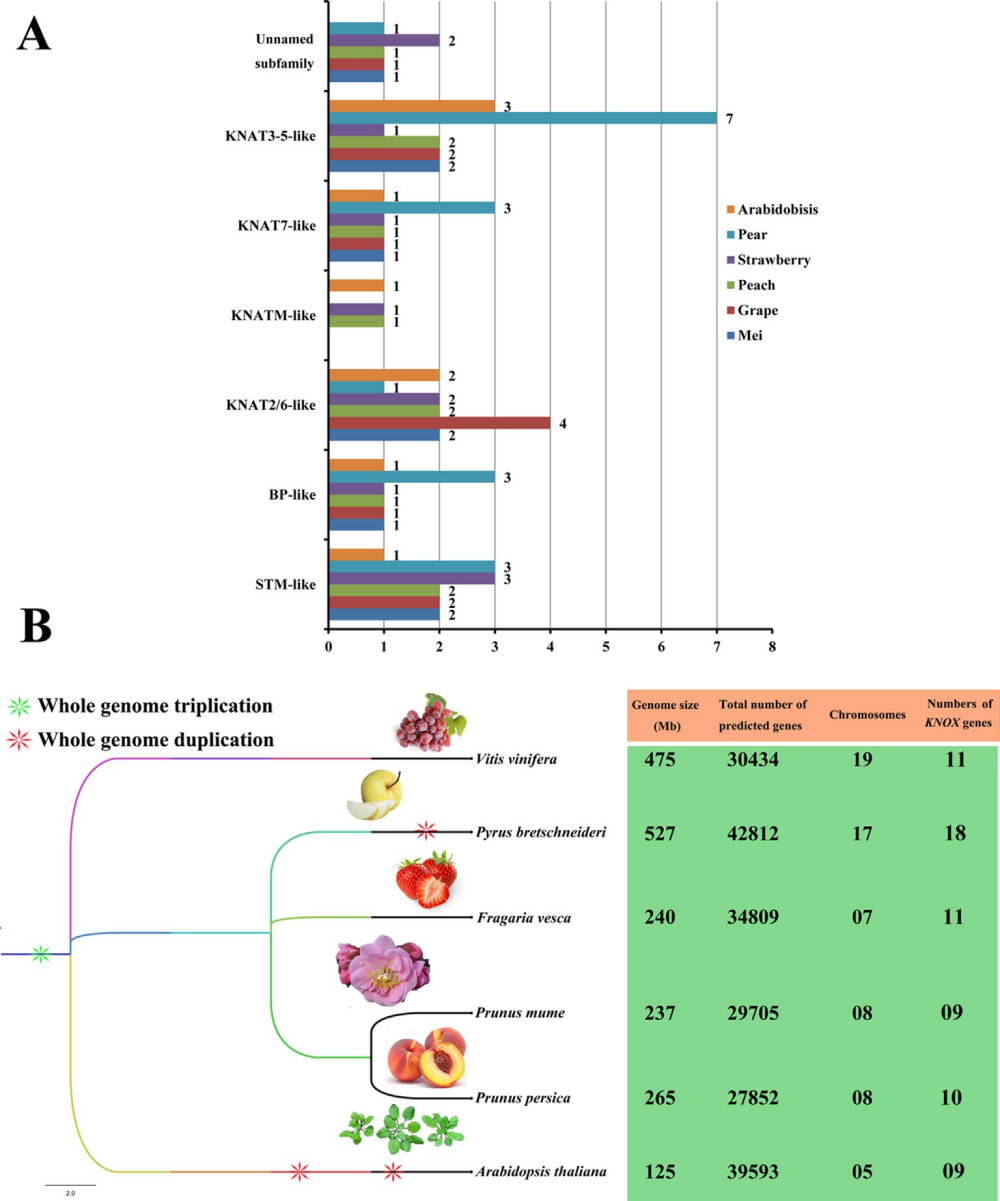
Subclade distributions and numbers of *KNOX* genes in different rosids. **(A)** Distributions of members of each subclade of the *KNOX* family from mei, grape, peach, strawberry, pear, and *Arabidopsis*. The number of KNOX proteins corresponding to each subclade is shown. **(B)** The species phylogenetic tree was obtained from Common Taxonomy Tree (https://www.ncbi.nlm.nih.gov/Taxonomy/CommonTree/wwwcmt.cgi) and was viewed in FigTree 1.4.3 (http://tree.bio.ed.ac.uk/).

We also constructed a species phylogenetic tree of six rosids (mei, grape, peach, strawberry, pear, and *Arabidopsis*) and performed statistical analyses on the numbers of *KNOX*s in each species (**Figure 3**). Among the six rosids, only peach, strawberry, and *Arabidopsis* have KNATM-like family members. For the *PbKNOX* family, the number of members in the BP-like, KNAT7-like, and KNAT3-5-like subfamilies is higher than those of other species. In addition, the numbers of *KNOX* family members are not positively related to genome size or the number of chromosomes in a species (**Figure 3**). For example, the genome size and chromosome number of grape are approximately double than those of strawberry, but the number of *KNOX*s is the same for each species. Interestingly, the numbers of *KNOX* genes per megabase (Mb) in mei (0.037), grape (0.02), peach (0.038), strawberry (0.045), and pear (0.03) were similar, ranging from 0.02 in grape to 0.038 in peach. These values were lower than that observed in *Arabidopsis* (0.072).

### Analyses of the *cis*-Acting Elements in *KNOX* Family Genes in Pear

**Figure 4:**
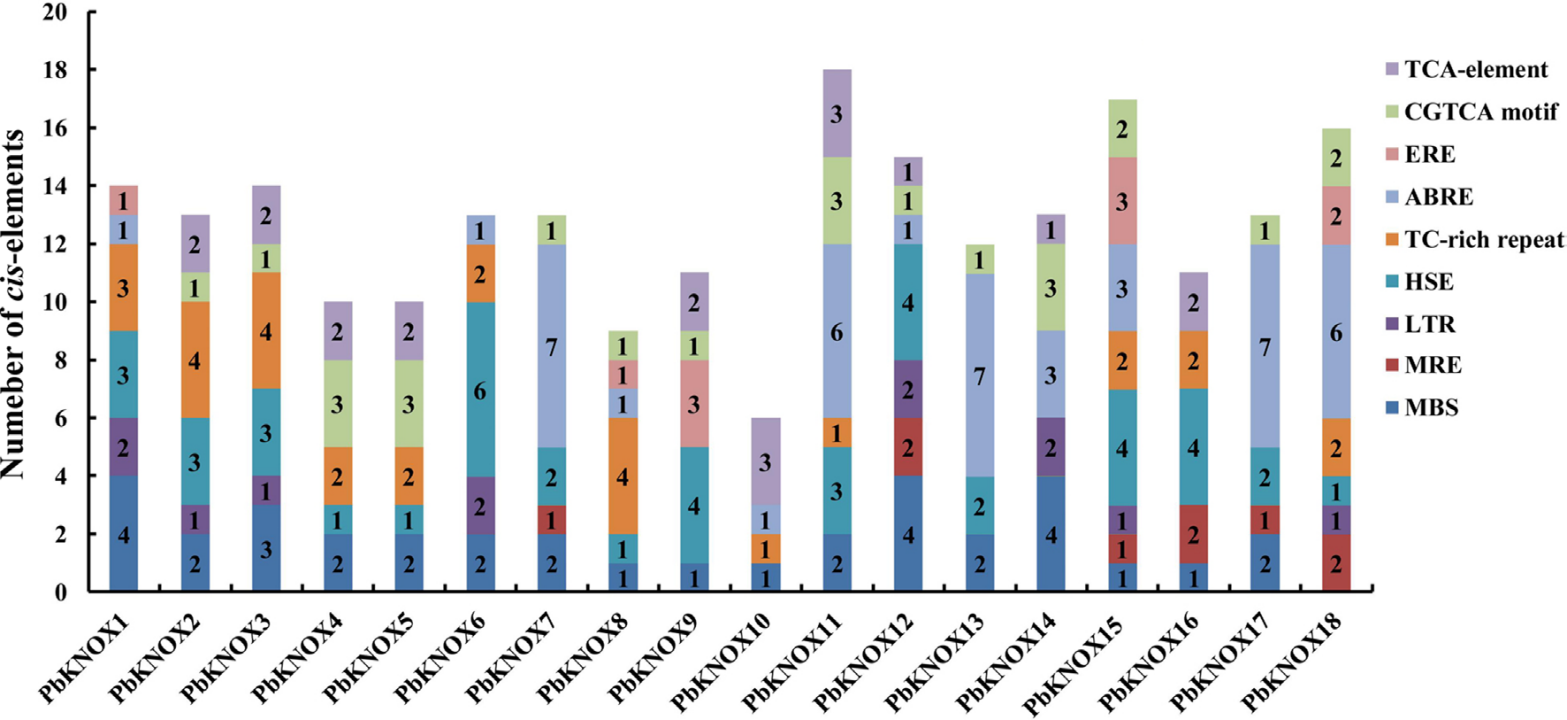
Putative *cis*-acting regulatory elements in the *PbKNOX* promoters.

We identified *cis*-acting elements in the 1,500-bp promoter sequence of each family member to obtain an understanding of the potential regulatory mechanisms underlying the expression of *PbKNOX* family members. The promoter regions of the 18 *PbKNOX* genes contained a large number of *cis*-acting elements associated with environmental stress and hormone responses. These elements include drought-related MBS elements, photoresponsive MRE elements, temperature stress-related HSE and LTR elements, and elements associated with abscisic acid (ABRE), ethylene (ERE), salicylic acid (TCA-element), and methyl jasmonate (CGTCA motif) responses (further details are provided in **Figure 4** and **Table S7**). These results suggest that the transcription of *PbKNOX* genes may be affected by these factors.

### Classifications and Predicted Functions of PbKNOX Proteins

**Figure 5:**
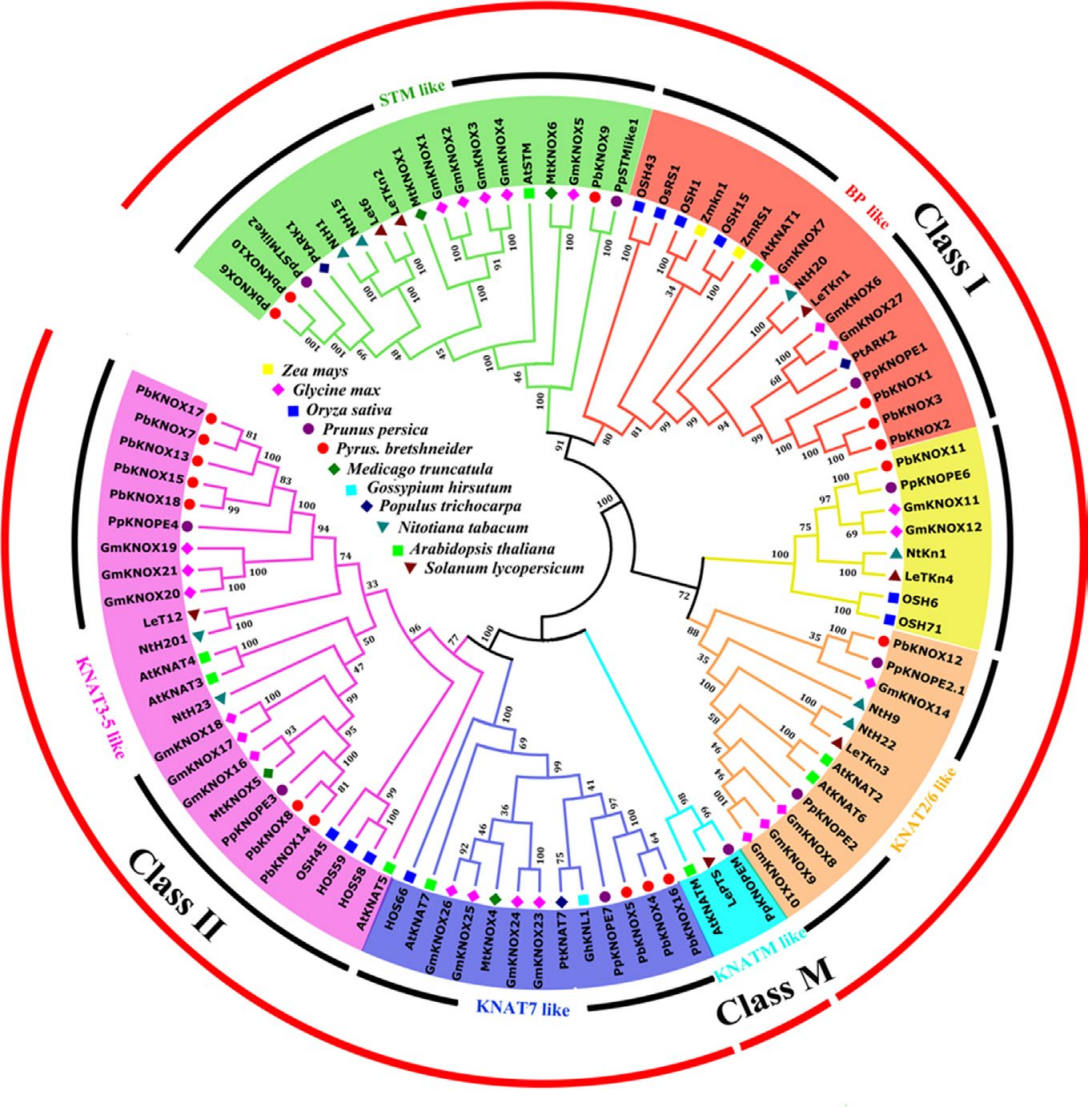
Interspecific phylogenetic tree of *KNOX* protein sequences from various species. Bootstrap values (at the branching points) are given for major nodes and are based on 1,000 replicates. Names of the species and the GenBank accession numbers of the sequences used in this tree are listed in **Table S1**.

We constructed an interspecies phylogenetic tree between PbKNOX proteins and other known KNOX proteins to further analyze the evolutionary relationships of pear KNOX proteins and to predict their possible biological functions. In this study, 96 sequences of KNOX proteins derived from *P. bretschneideri*, *O. sativa*, *Glycine max*, *A. thaliana*, *Solanum lycopersicum*, *Medicago truncatula*, *Nicotiana tabacum*, *Prunus persica*, *Gossypium hirsutum*, and *Z. mays* were used to construct interspecies phylogenetic trees (**Figure 5**).

As shown in **Figure 5**, KNOXs from different species were clearly divided into three classes, class I, class II, and class M. According to the classification method reported by Testone et al. (2012), these proteins were further divided into six subfamilies: class I includes STM-like, BP-like, and KNAT2/6-like subfamilies; class II consists of KNAT7-like and KNAT3-5-like subfamilies; and class M only contains the KNATM-like subfamily. All members of the pear *KNOX* family were distributed among classes I and II, and members of class M have not yet been identified. Among the pear KNOX proteins, PbKNOX1, 2, and 3 belong to the BP-like subfamily, while PbKNOX6, 9, and 10 belong to the STM-like subfamily. PbKNOX12 is a member of the KNAT2/6-like subfamily; PbKNOX4, 5, and 16 belong to the KNAT7-like subfamily, and PbKNOX7, 8, 13, 14, 15, 17, and 18 are classified as belonging to the KNAT3-5-like subfamily. There is also a clade in class I that is not named, and PbKNOX11 is present in the clade; the classification of the peach *KNOX* family is similar to that of the pear *KNOX* family (Testone et al., 2012).

Currently, class I KNOXs related to lignin metabolism and cell wall development have been shown to be derived from two subfamilies: BP-like or STM-like. In the phylogenetic tree constructed in this study, *Lycopersicon esculentum* T6 (LeT6) and *Populus* ARBORKNOX1 (ARK1) in the STM-like subfamily are involved in regulating the lignin composition or cell wall biosynthesis (Du et al., 2009; Townsley et al., 2013). In addition, *A. thaliana* STM has been shown to regulate the transcription of *AtKNAT1/BP* (Byrne et al., 2002; Scofield et al., 2014). PbKNOX6 and PbKNOX10 are grouped together with the same clade, suggesting that they may have similar biological functions.

AtKNAT1/BP, PpKNOPE1, *Populus* AKR2, maize Knotted1 (Kn1), maize Roughsheath1 (Rs1), and *O. sativa* OSH1, in the BP-like subfamily, have been shown to be involved in the negative regulation of lignification and/or secondary wall synthesis (Testone et al., 2012; Townsley et al., 2013; Yoon et al., 2017), and it has been suggested that the members of this subfamily are closely involved in plant cell wall development and lignification. PbKNOX1, PbKNOX2, and PbKNOX3 not only belong to this subfamily but also have close phylogenetic relationships with other known KNOX proteins, indicating that these three PbKNOX proteins may also function to negatively regulate pear lignin and cell wall biosynthesis.

The KNOX proteins in class II, AtKANT7, and GhKNL1, which belong to the KNAT7-like subfamily, are associated with secondary wall development and lignin metabolism (Li et al., 2012; Gong et al., 2014). PbKNOX4, PbKNOX5, and PbKNOX16 are clustered with AtKANT7 and GhKNL1, although further studies are needed to determine whether they have the same functions.

### Sequence Alignments of Pear KNOXs

Currently, AtBP, AtSTM, and AtKNAT7 in the *Arabidopsis KNOX* gene family have been widely studied (Mele et al., 2003; Li et al., 2012; Scofield et al., 2014), and their functions in regulating lignin metabolism have been relatively clearly defined. In addition, orthologues of AtBP, including PpKNOPE1, OSH1, ZmKn1, and PtAKR2; orthologues of AtSTM, including PtAKR1 and LeT6; and the orthologue of AtKNAT7 and GhKNL1 are involved in the mechanisms regulating lignin and cell wall biosynthesis. This study used the bioinformatics software Sequence Manipulation Suite (https://sites.ualberta.ca/∼stothard/javascript/ident_sim.html) to analyze the sequence similarities and identities of *PbKNOX* family members with these known KNOX proteins to identify candidate PbKNOX proteins related to lignin and cell wall synthesis in pears (**Table S8**).

The results showed that the 18 PbKNOX proteins did not show high sequence identity with either AtKNAT7 or GhKNL1, which is consistent with the clustering results in the phylogenetic tree (**Table S8** and **Figure 5**). The comparisons of the deduced PbKNOX6 and PbKNOX10 sequences with those of PtAKR1 and AtSTM showed 60–68% identity (Iden) and 68–84% similarity (Sim). Further studies are needed to verify whether these proteins have similar functions.

PbKNOX1 and PbKNOX2 (belonging to the BP-like subfamily) have exceptionally higher levels of similarly (70–91%) and identity (60%) to the AtBP than the other PbKNOX proteins. In addition, PbKNOX1 and PbKNOX2 have high sequence similarities and identities with the BPs in dicotyledonous plants, such as PpKNOPE1 (Iden/Sim 89%/94% and 89%/95%, respectively) and PtAKR2 (Iden/Sim 71%/85% and 71%/84%, respectively). Unexpectedly, PbKNOX3 did not share high sequence similarities and identities with these proteins (**Table S8**). Combined with the results of phylogenetic tree clustering, PbKNOX1 and PbKNOX2 are considered orthologues of AtBP in pears. These two PbKNOX proteins have high sequence similarities and identities with lignin/cell wall biosynthesis-related KNOX proteins, suggesting that they may have similar biological functions.

### Expression Profiling of Pear *KNOX* Family Members

**Figure 6:**
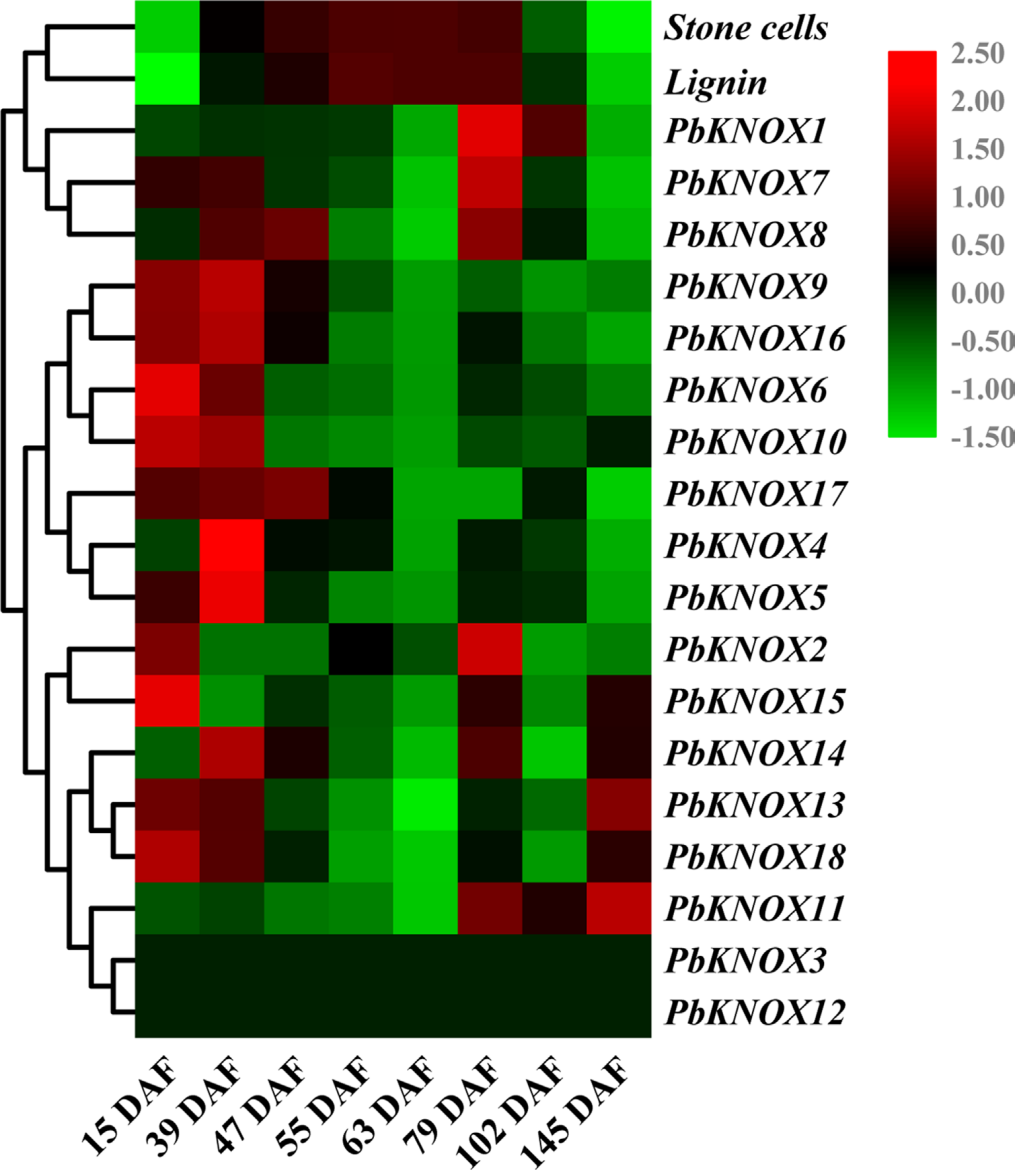
Hierarchical clustering analysis of stone cell/lignin levels, and transcription levels of *PbKNOX*s in pear fruits at different developmental stages. The qRT-PCR results are presented in a heatmap, and the expression trends were clustered. Stone cells: percentage of stone cell content in pear pulp (%). Lignin: percentage of lignin content in pear pulp (%).

In this study, we analyzed the temporal and spatial expression patterns of pear *KNOX* family members and further explored their relationships with stone cell development and lignin synthesis (**Figure 6** and **Figure S6**).

The expression profiles of the 18 *KNOX* genes in pear fruit showed that *PbKNOX4*, *5*, *6*, *9*, *10*, *16*, and *17* were clustered into one class. Higher levels of these seven transcripts were observed in the early stage of fruit development, and low levels of the transcripts were observed during the middle and later stages of fruit development. The expression of *PbKNOX13*, *14*, *15*, and *18* peaked at 15 DAF, 23 DAF, 79 DAF, and at maturity, respectively, and lower levels of these transcripts were observed during the other stages of fruit development. In addition, *PbKNOX11* was expressed only in the late stage (79 DAF to maturity) of fruit development (**Figure 6**). 

The expression levels of *PbKNOX1*, *2*, *7*, and *8* have peaks at 79 DAF, but *PbKNOX2*, *7*, and *8* also have peaks between 15 DAF and 47 DAF, while *PbKNOX1* has lower transcript abundance before 79 DAF. According to the qRT-PCR results, *PbKNOX3* and *PbKNOX12* were not detected in different developmental stages of pear fruit and were not expressed in certain tissues, and it was speculated that these two *KNOX* genes did not play roles in these organs (**Figure 6** and **Figure S6**). The other 16 *PbKNOX* genes were expressed at higher levels in at least one of the tissues, including buds, stems, leaves, and flowers, and did not exhibit significant tissue specificity (**Figure S6**).

As shown in **Figure 6**, the contents of stone cells and lignin in pear fruit showed a rise-fall tendency, with the relative content beginning to increase after 15 DAF and beginning to decrease after 79 DAF. Notably, previous studies have shown that, although the levels of stone cells and lignin are lower near 15 DAF, at this point, the secondary cell-wall thickening and lignification have already begun, and the transcription of related genes has been initiated (Zhao et al., 2013; Cheng et al., 2017; Xue et al., 2018). This finding indicates that 15 DAF to 79 DAF is a vigorous synthesis period for pear stone cells and lignin. Based on phylogenetic tree clustering and sequence alignment analyses, the expression pattern of *PbKNOX1* showed an opposite tendency to the pattern of stone cell and lignin content, suggesting that *PbKNOX1* is a potential inhibitor of pear lignin metabolism and cell wall development. 

In light of the results of the phylogenetic tree, sequence alignment, and expression pattern analyses, *PbKNOX1* was selected as a candidate gene for studying its role in lignin metabolism and cell wall development.

### Effects of the Heterologous Expression of *PbKNOX1* on Cell Wall Development and Lignification in *Arabidopsis thaliana*

We cloned the full length CDS of *PbKNOX1* and named it *PbKNOX1/BP*. A eukaryotic expression vector containing *PbKNOX1/BP* was constructed and overexpressed in *Arabidopsis* (**Figure S7** and **Figure S8**). Three homozygous *PbKNOX1/BP* transgenic lines (PbBP-1, PbBP-2, and PbBP-3) were selected for further analyses to confirm the functions of *PbKNOX1/BP* in cell wall development and lignification. 

**Figure 7:**
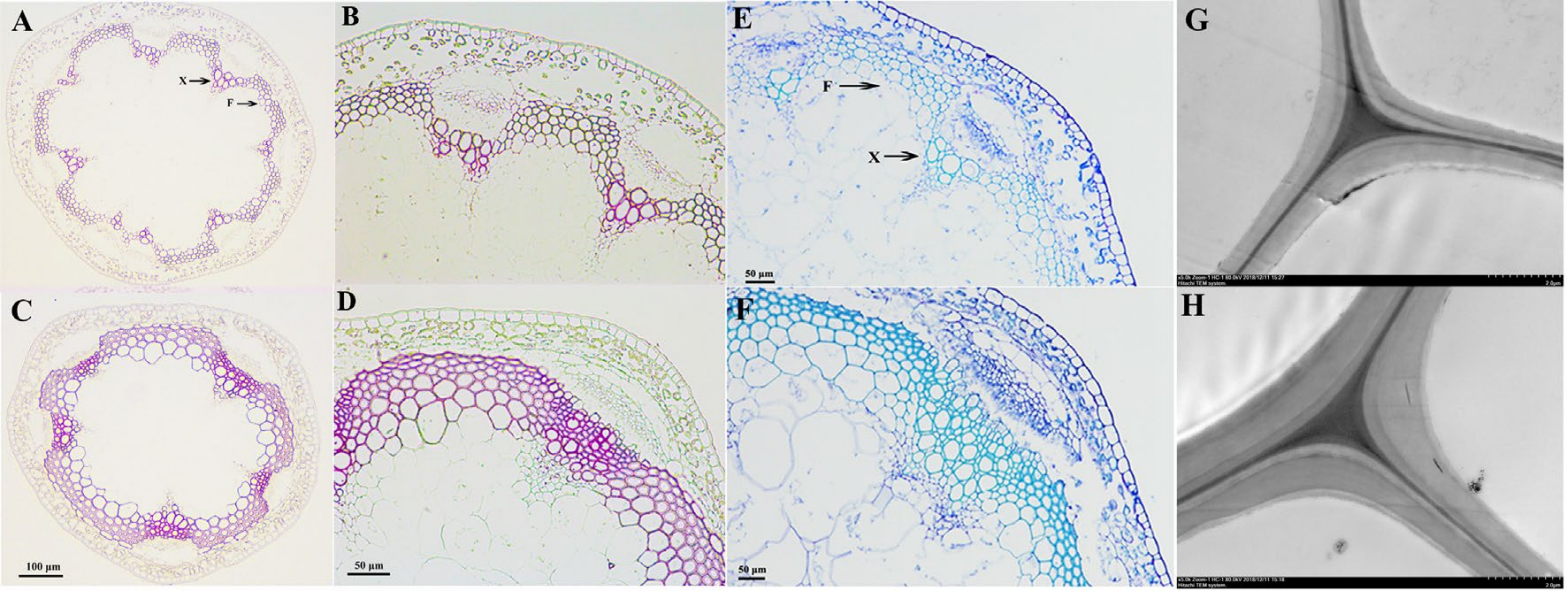
Microscopic and ultramicroscopic observation of cell walls in the inflorescence stems of WT and *PbBP*-overexpressing transgenic lines. **(A**, **B**, **E**, and **G)** *PbBP*-overexpressing transgenic lines; **(C**, **D**, **F**, and **H)** wild-type *Arabidopsis* (WT); F: interfascicular fiber; X: xylem. **(A**, **B**, **C**, and **D)** Wiesner staining (lignin deposition patterns) of cross-sections of *Arabidopsis* inflorescence stem; **(B** and **D)** show higher magnification images of **(A)** and **(C)**, respectively. **(E** and **F)** Toluidine blue staining of the inflorescence stems from WT and transgenic lines; **(G** and **H)** TEM images of the ultrastructure of the cell wall.

We used toluidine blue staining, Wiesner staining, and TEM to visually observe the effects of *PbKNOX1/BP* on the morphological characteristics of developing cell walls in the inflorescence stem of wild-type and transgenic *Arabidopsis* (**Figure 7**). Wiesner and toluidine blue staining results showed that, compared with wild-type *Arabidopsis*, the transgenic plants not only had reduced xylem and interfascicular fiber regions but also had a significantly reduced degree of staining (**Figures 7A–F**). This result indicates that the deposition of lignin in the cell walls of the transgenic plants was less than that of wild-type plants. Notably, toluidine blue staining and TEM observations revealed a significantly reduced thickness of the cell walls in the transgenic plants compared with the wild-type plants (**Figures 7F–H**). By measuring the cell wall thickness in the TEM image, it was found that the cell wall thickness of the transgenic plants decreased by approximately 19% compared with that of the wild-type plants (**Figure S9**). These results suggest that *PbKNOX1/BP* could be involved in negative regulation of plant cell wall development and lignification.

### Overexpression of *PbKNOX1* Resulted in a Decrease in the Lignin Content of Transgenic Plants

**Figure 8:**
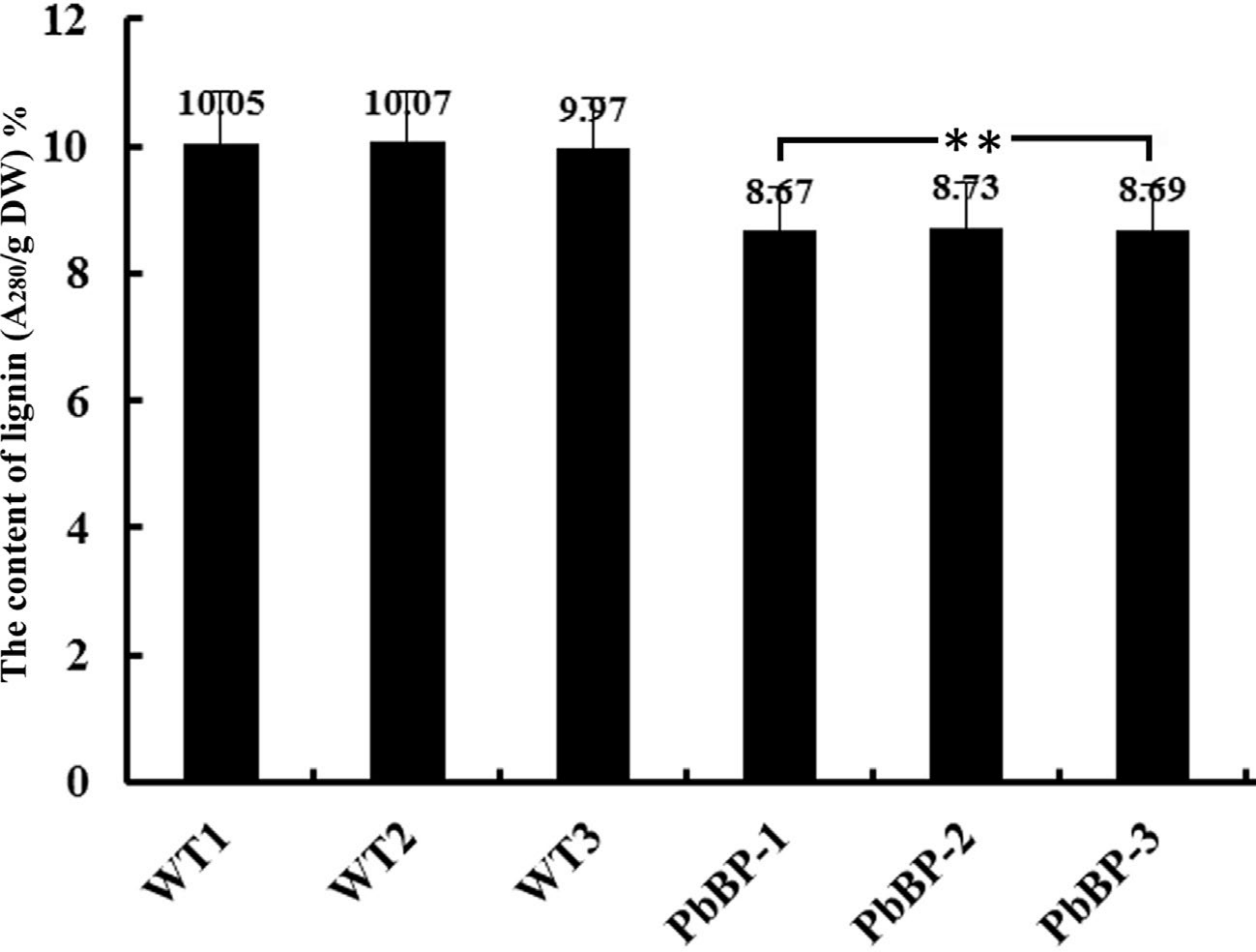
Lignin content in *PbKNOX1/BP* transgenic lines. WT-1∼3: wild-type *Arabidopsis*; PbBP-1∼3: *PbBP*-overexpressing transgenic lines. **Significant difference between the lignin content of the WT and transgenic plants (*p* < 0.01).

Previous studies have found that *BP* inhibits the biosynthesis of lignin. In this study, the acetyl bromide method was used to determine the lignin content in the inflorescence stems of wild-type *Arabidopsis* (WT-1, WT-2, and WT-3) and transgenic *Arabidopsis* (PbBP-1, PbBP-2, and PbBP-3) plants. The results showed that the lignin content in the transgenic lines decreased by approximately 13% compared with that of the wild-type *Arabidopsis* lines, and the difference reached an extremely significant level (*p* < 0.01) (**Figure 8**). This result indicates that *PbKNOX1/BP* overexpression can hinder the biosynthesis of lignin in plants.

**Figure 9:**
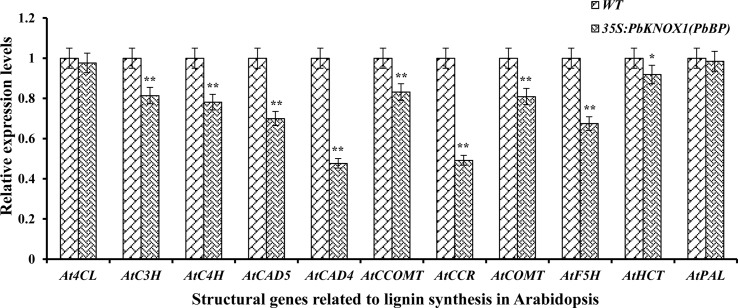
The expression of eleven lignin genes was profiled by qRT-PCR in the inflorescence stem of the wild-type and *PbBP*-overexpressing transgenic lines. WT: wild-type *Arabidopsis*; *35S::PbKNOX/BP*: *PbBP*-overexpressing transgenic lines. **p < 0.01; *p < 0.05.

The levels of transcripts encoded by an 11 gene set (covering the early, intermediate, and late steps of lignin biosynthesis) were monitored in the stem inflorescence to further investigate the role of PbKNOX1/BP in the lignin pathway (**Figure 9**). As shown in **Figure 9**, the overexpression of *PbKNOX1/BP* in *Arabidopsis* significantly inhibited the expression of the key structural genes involved in lignin metabolism. These genes include the following: *AtC4H*, which is located in the general phenylpropanoid pathway; *AtC3H*, *AtHCT*, and *AtCCOMT*, which are responsible for the ester intermediary pathway; *AtCCR*, *AtCAD4*, and *AtCAD5*, which catalyze the monolignol-specific biosynthesis pathway; and *AtF5H* and *AtCOMT*, which are involved in monolignols conversion. The results suggest that *C4H*, *C3H*, *HCT*, *CCOMT*, *CCR*, *F5H*, *COMT*, and *CAD* may be target genes of *PbKNOX1/BP*.

### Discussion

The stone cells of pear are a type of sclerenchyma cell (SCW forming cell), and the thickened SCW completely fills the cell lumen and is highly lignified (Jin et al., 2013; Barros et al., 2015). Excessive stone cells can cause inferior pear fruit flavor and gritty texture (Choi et al., 2007; Xue et al., 2018). Therefore, regulating stone cell development is of great significance for improving the quality of pear fruit. The *KNOX* family has been shown to play a negative regulatory role in lignin metabolism and cell wall development (Li et al., 2012; Townsley et al., 2013). However, studies on the *KNOX* family in pears have rarely been reported. However, studies of the *KNOX* family in pears have rarely been reported. Therefore, we identified genes related to lignin and cell wall biosynthesis from the *PbKNOX* family and performed functional analyses. This study lays a foundation for the future regulation of pear lignin synthesis and stone cell development.

In this study, we identified 18 *PbKNOX*s at the whole genome level of pear. In addition, we found that there are nine *KNOX*s in the grape genome, while 11 *KNOX*s were identified in both the mei and strawberry genomes (**Table S5**). Previous studies have suggested that the *KNOX* gene family may have a limited number of members according to the genome size (Testone et al., 2012). For example, in contrast to apples, the genomes of *Arabidopsis*, peach, rice, maize, and poplar have no more than 15 *KNOX* family members (Li et al., 2012; Testone et al., 2012). Because coding theory predicts an upper limit for the number of TFs, cross-binding errors between TFs are minimized (Itzkovitz et al., 2006). However, the number of *KNOX* family members was not simply positively correlated with the genome size in the present study (**Figure 3**). Apples and pears have the closest relationship (Wu et al., 2013), and more than 15 *KNOX* genes have been identified in the two species. Researchers have speculated that the expansion of the *KNOX* family in apple and pear may be related to their species divergence.

Interestingly, conserved domain analyses showed that PbKNOX4, 5, and 16 lacked the ELK and Homeobox_KN domains (**Figure 1**), which have the same characteristics as KNATM (Gao et al., 2015). However, based on the cluster analyses, pear does not contain members of the KNATM-like subfamily, and all three PbKNOX proteins lacking these domains (PbKNOX4, 5, and 16) belong to the KNAT7-like subfamily (**Figure 5**). Moreover, no similar situation was found in the *Arabidopsis* and peach *KNOX* families. Therefore, the absence of the ELK and Homeobox_KN domains may not be unique features of KNATM. It is also possible that PbKNOX4, 5, and 16 lost the ELK and Homeobox_KN domains during evolution.

An analysis of *cis*-acting elements revealed a large number of elements related to drought and heat stress in most of the *PbKNOX* promoters (**Table S7** and **Figure 4**). Previous studies have found that the lack of water in the soil leads to an increase in the content and density of stone cells in pear fruits (Lee et al., 2006). It has been speculated that the *PbKNOX* family may be involved in this process. In addition, the *PbKNOX* promoters also contain hormone-responsive elements, such as the TCA element, CGTCA motif, and ABRE motif. Thus, the expression of the *PbKNOX* family may be regulated by these plant hormones. Although the effects of ABA and MeJA on the development of pear stone cells have not been reported, SA regulates lignin synthesis and stone cell development in pear fruit by inducing the expression of microRNAs (Xue et al., 2018). We speculate that these three hormones may also directly or indirectly regulate the expression of *PbKNOX*s, thereby affecting stone cell formation. In the future, we plan to systematically study the effects of water stress and exogenous hormones on the transcription of *PbKNOX*s to determine whether the development of pear cells can be controlled by regulating these factors in field production. 

The spatiotemporal expression analyses of *PbKNOX* family members in pears showed more conservative expression patterns of STM-like subfamily gene members (*PbKNOX6*, *9*, and *10*), and the genes were primarily expressed in fruits (at the early stage of development) and flowers (**Figure 6** and **Figure S6**). Through phylogenetic tree clustering and sequence alignments, *PbKNOX6* and *10* were not only located in the same clades as the known STMs from other species but also displayed high sequence similarity and consistency (57–84%) (**Figure 5** and **Table S8**). Therefore, we speculate that *PbKNOX6* and *10* are the *STM* genes in pear. Previous studies have shown that *STM* can inhibit the expression of *BP* (Byrne et al., 2002). In the present study, the expression of *PbKNOX1/BP*, a member of the BP-like subfamily, showed opposite trends to *PbKNOX6* and *10* (**Figure 6**), suggesting that similar regulatory mechanisms may be employed by these genes.

The expression pattern of *PbKNOX2* is different from that of *PbKNOX1/BP*, and *PbKNOX2* has a higher transcription level at the early stage of pear fruit development (**Figure 6**). Although the content of stone cells and lignin is low in early developmental fruit (**Figure 6**), at this time (15 DAF), the primordial cells of the stone cells have been formed, and the secondary thickening and lignification of the cell wall has begun (Cai et al., 2010; Jin et al., 2013; Zhao et al., 2013; Xue et al., 2018). Therefore, the expression of lignin metabolism related genes must be at a high level at this time to synthesize sufficient lignin monomer as the raw material for secondary wall thickening (Xue et al., 2018). *PbKNOX2*, which is more similar to the *PbKNOX1* sequence, has higher expression levels at 15 DAF. Currently, there is no research to prove that KNOX has the function of positively regulating lignin synthesis. Therefore, the specific function of *PbKNOX2* requires further study.

Importantly, our analysis indicates that *PbKNOX1/BP* plays an important role in cell wall development and lignin biosynthesis. Overexpression of *PbKNOX1/BP* in wild-type *Arabidopsis* resulted in thinner xylem and interfascicular fiber cell walls. The total lignin content in the inflorescence stem of *PbKNOX1/BP*-overexpressing transgenic *Arabidopsis* was decreased by 13% compared to wild-type *Arabidopsis* (**Figures 7** and **8**). The orthologues of the *Arabidopsis BP* gene, such as *PpKNOPE1*, *ZmKN1*, *OSH15*, and *PtARK2*, have similar functions in dicotyledonous and monocotyledonous plants (Du et al., 2009; Testone et al., 2012; Townsley et al., 2013; Yoon et al., 2017). Therefore, we speculate that the orthologous genes of *AtBP* are functionally conserved in different species. The use of a fruit-specific promoter to drive *PbKNOX1/BP* overexpression in pear fruit may inhibit the formation of stone cells to some extent, thus improving fruit quality.

**Figure 10:**
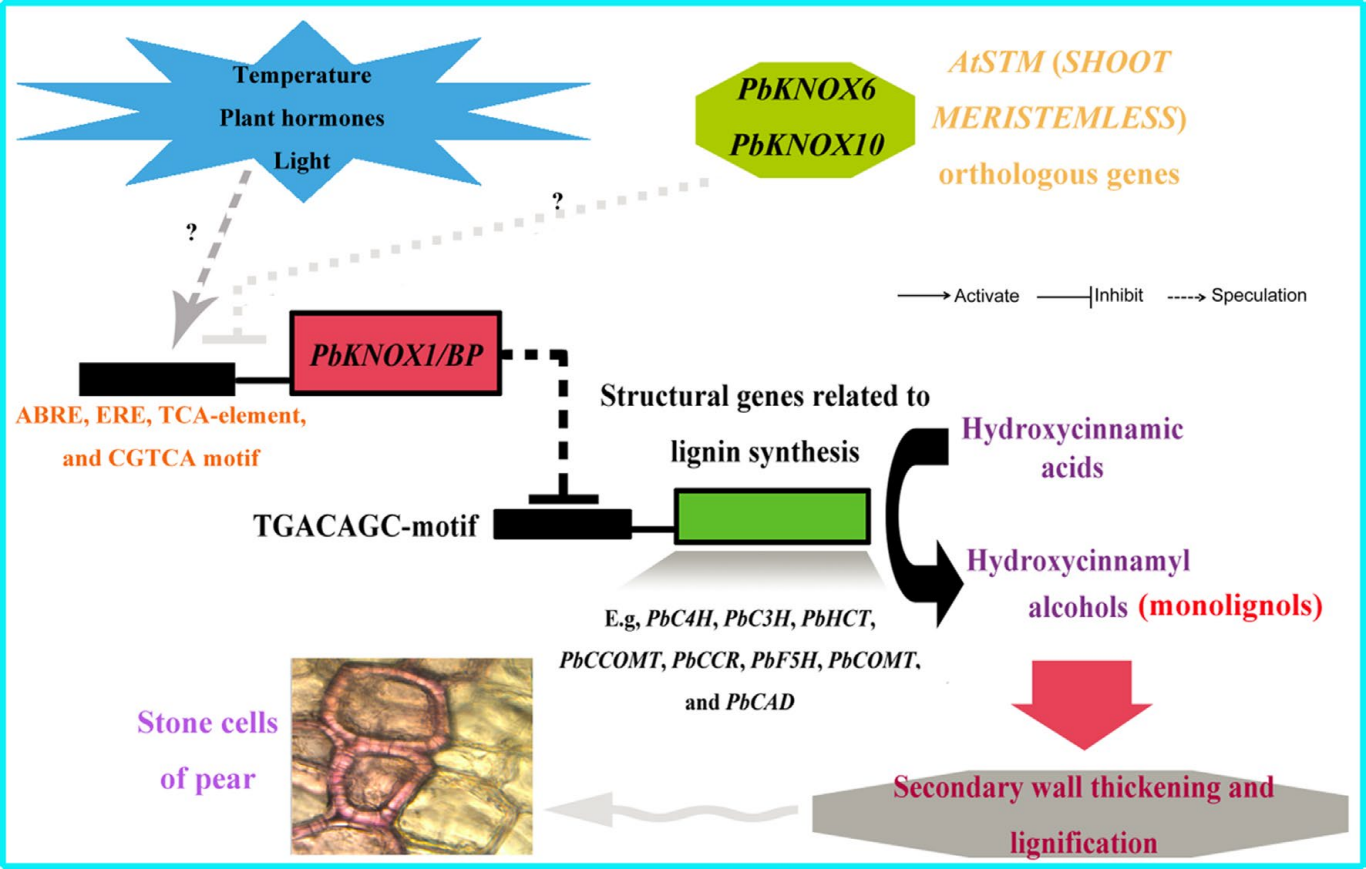
Model depicting the proposed regulatory interactions of *PbKNOX1/BP* on pear lignin metabolism and stone cell development. T bar and arrow refer to negative and positive effect on downstream effector or biological process, respectively.

More strikingly, *PbKNOX1/BP* can also reduce the transcription levels of multiple structural genes in the lignin metabolic pathway (**Figure 9**). The expression levels of *C4H*, *C3H*, *HCT*, *CCOMT*, *CCR*, *F5H*, *COMT*, and *CAD*, which are key genes for lignin synthesis, were significantly reduced in *PbKNOX1/BP*-overexpressing *Arabidopsis*. Our findings are consistent with previous studies on poplar *ARK2*, peach *KNOPE1*, and *Arabidopsis BP* (Mele et al., 2003; Du et al., 2009; Testone et al., 2012). *KNOX* may regulate cell wall development and lignification primarily by inhibiting the lignin metabolic pathway. Notably, studies in *Arabidopsis* and peach have shown that *AtBP* and *PpKNOPE1* can interact with the typical KNOX DNA-binding site (TGACAGC-motif) to regulate the expression of key structural genes involved in lignin metabolism (Mele et al., 2003; Testone et al., 2012). We have now identified potential TGACAGC motifs in the promoters of 10 pear lignin metabolism-related genes, such as *PbCCOMT* (GenBank no. KX500357) and *PbCOMT* (GenBank no. KX500356) (**Table S9**). Therefore, a focus of our future studies is to determine whether the same regulatory mechanism exists in pear. Finally, based on the findings of this study and some previous conclusions, a model of the regulatory effects of PbKNOX1/BP on pear cell development and lignin metabolism was proposed (**Figure 10**).

## Conclusions

In the present study, 18 non-repetitive *KNOX* genes were identified in the pear genome, which were primarily distributed among STM-like, BP-like, KNAT2/6-like, KNAT7-like, and KNAT3-5-like subfamilies. Phylogenetic tree clustering and sequence alignments indicated that *PbKNOX1* is a pear orthologue of the *Arabidopsis BP* gene. *PbKNOX1* expression was inversely correlated with pear stone cell lignification. Heterologous expression of* PbKNOX1* in *Arabidopsis* revealed that this gene not only significantly inhibited cell wall thickening and lignification but also significantly reduced the plant lignin content. Our research provides a potential new strategy for regulating pear stone cell development.

## Author Contributions

XC, ML, and MA performed the experiments and wrote the paper. XC and ML analyzed the data. GL and JZ helped process the data. MA and MAM helped to polish the language. JZ, HW and QJ contributed reagents and materials. XC, TJ, and YC discussed and analyzed the results. YC, YL, and DL conceived and designed the experiments. All authors read and approved the final manuscript.

## Funding

This study was supported by the National Natural Science Foundation of China (grant #31640068) and Anhui Provincial Natural Science Foundation (grant # 1808085MC58 and grant # 1808085QC79).

## Conflict of Interests Statement

The authors declare that the research was conducted in the absence of any commercial or financial relationships that could be construed as a potential conflict of interest.

## Supplementary Material

Supplementary Figure 1 The exon–intron structure and conserved motifs of *PbKNOX*s based on the evolutionary relationship. **(A)** Phylogenetic tree of PbKNOX family members. **(B)** Conserved motifs of *PbKNOX* family members. **(C)** The exon-intron structure of *PbKNOX* family members.

Supplementary Figure 2 Multiple sequence alignment of PbKNOX12 with its orthologs.

Supplementary Figure 3 Chromosomal locations and gene duplications of the *PbKNOX*s on the ten chromosomes. Genes involved in segmental duplication are joined by dashed lines, and the red lines indicate gene clusters. The number corresponding to each gene name represents its position along the chromosome, and the number corresponding to each chromosome name represents the total length of the chromosome.

Supplementary Figure 4 Interspecies phylogenetic tree, gene structures and conserved motifs of *KNOX* family members in four rosids. **(A)** Phylogenetic tree of *KNOX* family members in four rosids. **(B)** The exon–intron structure of *KNOX* family members. **(C)** Conserved motifs of *KNOX* family members.

Supplementary Figure 5 Microsynteny regions of *KNOXs* among *Pyrus bretschneideri*, *Fragaria vesca*, *Prunus mume* and *Vitis vinifera*. The green bars represent chromosomes, and there are chromosomes types and regions on the right. The numbers on both sides of the chromosome are the suffixes of each gene Genome ID. Homologous genes pairs is connected by a straight line, except that *KNOX* is red, and other homologous genes is expressed in blue, and non-homologous genes is expressed in white.

Supplementary Figure 6 Expression patterns of *PbKNOX*s in pear in different tissues. In this study, *Tubulin* (accession no. AB239680.1) was used as an internal reference. Each qRT-PCR analysis was performed in triplicate. 15 DAF: 15 DAF pear fruits.

Supplementary Figure 7 Acquisition and confirmation of transgenic Arabidopsis seedlings. **(A)** Obtaining hygromycin-resistant Arabidopsis seedlings; **(B)** The GUS staining of transgenic Arabidopsis seedling; **(C)** The GUS staining of wild-type Arabidopsis seedling; **(D)** Construction of the eukaryotic expression vector pCAMBIA1304-*PbKNOX1*.

Supplementary Figure 8 Sequence alignment of PbKNOX/BP and other lignin/cell wall biosynthesis-related KNOXs.

Supplementary Figure 9 Statistical analysis of the secondary cell wall thickness of vessel cells in WT and *PbKNOX1/BP*-overexpressing transgenic plants. **Significant difference between the secondary cell wall thickness of the WT and transgenic plants (*P*< 0.01). WT: Wild-type Arabidopsis; PbBP-1∼3: *PbKNOX1/BP*-overexpressing transgenic lines.

Supplementary Table 1 GenBank accession codes used for constructing phylogenetic trees.

Supplementary Table 2 Primers for qRT-PCR and vector construction.

Supplementary Table 3 Details of 20 conserved motifs in the PbKNOXs.

Supplementary Table 4 Ka/Ks analysis for *KNOX* duplicated genes of pear.

Supplementary Table 5 List of *KNOX* protein sequences of four rosids.

Supplementary Table 6 Collinearities were identified in four rosids.

Supplementary Table 7 Putative *cis*-acting regulatory elements in the *PbKNOX* promoters.

Supplementary Table 8 Sequence identity and similarity among PbKNOXs and *KNOX* protein sequences of various plants.

Supplementary Table 9 Potential KNOX binding sites in pear lignin genes.

## References

[B1] Abdullah M. Cao Y. Cheng X. Shakoor A. Su X. Gao J. (2018). Genome-wide analysis characterization and evolution of SBP Genes in *Fragaria vesca, Pyrus bretschneideri, Prunus persica and Prunus mume* . Front. Genet. 9, 1–12. 10.3389/fgene.2018.00064 29552026 PMC5841269

[B2] Anderson N. A. Tobimatsu Y. Ciesielski P. N. Ximenes E. Ralph J. Donohoe B. S. (2015). Manipulation of guaiacyl and syringyl monomer biosynthesis in an Arabidopsis cinnamyl alcohol dehydrogenase mutant results in atypical lignin biosynthesis and modified cell wall structure. Plant Cell 27, 2195–2209. 10.1105/tpc.15.00373 26265762 PMC4568507

[B3] Barros J. Serk H. Granlund I. Pesquet E. (2015). The cell biology of lignification in higher plants. Ann. Bot. 115, 1053–1074. 10.1093/aob/mcv046 25878140 PMC4648457

[B4] Brahem M. Renard C. M. G. C. Gouble B. Bureau S. Le Bourvellec C. (2017). Characterization of tissue specific differences in cell wall polysaccharides of ripe and overripe pear fruit. Carbohydr. Polym. 156, 152–164. 10.1016/j.carbpol.2016.09.019 27842809

[B5] Byrne M. E. Simorowski J. Martienssen R. A. (2002). ASYMMETRIC LEAVES1 reveals knox gene redundancy in Arabidopsis. Development 129, 1957–1965. 10.1007/978-3-642-36309-2 11934861

[B6] Cai Y. Li G. Nie J. Lin Y. Nie F. Zhang J. (2010). Study of the structure and biosynthetic pathway of lignin in stone cells of pear. Sci. Hortic. (Amsterdam). 125, 374–379. 10.1016/j.scienta.2010.04.029

[B7] Cheng X. Li G. Muhammad A. Zhang J. Jiang T. Jin Q. (2019). Molecular identification, phylogenomic characterization and expression patterns analysis of the *LIM* (*LIN-11*, *Isl1* and *MEC-3* domains) gene family in pear (*Pyrus bretschneideri*) reveal its potential role in lignin metabolism. Gene 686, 237–249. 10.1016/j.gene.2018.11.064 30468911

[B8] Cheng X. Li M. Li D. Zhang J. Jin Q. Sheng L. (2017). Characterization and analysis of *CCR* and *CAD* gene families at the whole-genome level for lignin synthesis of stone cells in pear (*Pyrus bretschneideri*) fruit. Biol. Open 6, 1602–1613. 10.1242/bio.026997 29141952 PMC5703608

[B9] Cheng X. Su X. Muhammad A. Li M. Zhang J. Sun Y. (2018). Molecular characterization, evolution, and expression profiling of the dirigent (DIR) family genes in Chinese white pear (*Pyrus bretschneideri*). Front. Genet. 9, 1–15. 10.3389/fgene.2018.00136 29713336 PMC5911567

[B10] Cheng X. Xiong Y. Li D. H. Cheng J. Cao Y. P. Yan C. C. (2016). Bioinformatic and expression analysis of the *OMT* gene family in Pyrus bretschneideri cv. Genet. Mol. Res. 15, 1–17. 10.4238/gmr.15038664 27706700

[B11] Choi J. Choi J. Hong K. Kim W. (2007). Cultivar Differences of Stone Cells in pear flesh and their effects on fruit quality. Hortic. Environ. Biotechnol. 48, 17–31.

[B12] Clough S. J. Bent A. F. (1998). Floral dip: a simplified method for Agrobacterium-mediated transformation of *Arabidopsis thaliana* . Plant J. 16, 735–743. 10.1046/j.1365-313x.1998.00343.x 10069079

[B13] Du J. Mansfield S. D. Groover A. T. (2009). The Populus homeobox gene ARBORKNOX2 regulates cell differentiation during secondary growth. Plant J. 60, 1000–1014. 10.1111/j.1365-313X.2009.04017.x 19737362

[B14] Frangedakis E. Saint-Marcoux D. Moody L. A. Rabbinowitsch E. Langdale J. A. (2017). Nonreciprocal complementation of *KNOX* gene function in land plants. New Phytol. 216, 591–604. 10.1111/nph.14318 27886385 PMC5637896

[B15] Furumizu C. Alvarez J. P. Sakakibara K. Bowman J. L. (2015). Antagonistic roles for *KNOX1* and *KNOX2* genes in patterning the land plant body plan following an ancient gene duplication. PLoS Genet. 11, 1–24. 10.1371/journal.pgen.1004980 PMC4335488 25671434

[B16] Gao J. Yang X. Zhao W. Lang T. Samuelsson T. (2015). Evolution, diversification, and expression of KNOX proteins in plants. Front. Plant Sci. 6, 1–12. 10.3389/fpls.2015.00882 26557129 PMC4617109

[B17] Gong S. Y. Huang G. Q. Sun X. Qin L. X. Li Y. Zhou L. (2014). Cotton KNL1, encoding a class II KNOX transcription factor, is involved in regulation of fibre development. J. Exp. Bot. 65, 4133–4147. 10.1093/jxb/eru182 24831118 PMC4112624

[B18] Itzkovitz S. Tlusty T. Alon U. (2006). Coding limits on the number of transcription factors. BMC Genomics 7, 1–15. 10.1186/1471-2164-7-239 16984633 PMC1590034

[B19] Jin Q. Yan C. Qiu J. Zhang N. Lin Y. Cai Y. (2013). Structural characterization and deposition of stone cell lignin in Dangshan Su pear. Sci. Hortic. (Amsterdam). 155, 123–130. 10.1016/j.scienta.2013.03.020

[B20] Lee T. H. Tang H. Wang X. Paterson A. H. (2013). PGDD: a database of gene and genome duplication in plants. Nucleic Acids Res. 41, 1152–1158. 10.1093/nar/gks1104 PMC3531184 23180799

[B21] Lee S. H. Choi J. H. Kim W. S. Han T. H. Park Y. S. Gemma H. (2006). Effect of soil water stress on the development of stone cells in pear (*Pyrus pyrifolia* cv. Sci. Hortic. (Amsterdam). 110, 247–253. 10.1016/j.scienta.2006.07.012

[B22] Li E. Bhargava A. Qiang W. Friedmann M. C. Forneris N. Savidge R. A. (2012). The Class II KNOX gene *KNAT7* negatively regulates secondary wall formation in Arabidopsis and is functionally conserved in Populus. New Phytol. 194, 102–115. 10.1111/j.1469-8137.2011.04016.x 22236040

[B23] Lin Y. X. Jiang H. Y. Chu Z. X. Tang X. L. Zhu S. W. Cheng B. J. (2011). Genome-wide identification, classification and analysis of heat shock transcription factor family in maize. BMC Genomics 12, 76. 10.1186/1471-2164-12-76 21272351 PMC3039612

[B24] Livak K. J. Schmittgen T. D. (2001). Analysis of relative gene expression data using real-time quantitative PCR and the 2^-DDCT^ method. Methods 25, 402–408. 10.1006/meth.2001.1262 11846609

[B25] Ma C. Zhang H. Li J. Tao S. Qiao X. Korban S. S. (2017). Genome-wide analysis and characterization of molecular evolution of the HCT gene family in pear (*Pyrus bretschneideri*). Plant Syst. Evol. 303, 71–90. 10.1007/s00606-016-1353-z

[B26] Mele G. Ori N. Sato Y. Hake S. (2003). The knotted1-like homeobox gene *BREVIPEDICELLUS* regulates cell differentiation by modulating metabolic pathways. Genes Dev. 17, 2088–2093. 10.1101/gad.1120003 12923061 PMC196451

[B27] Pradhan Mitra P. Loqué D. (2014). Histochemical staining of Arabidopsis thaliana secondary cell wall elements. J. Vis. Exp. 2014 (87), 51381. 10.3791/51381 PMC4186213 24894795

[B28] Reveal J. L. Chase M. W. (2011). APG III: bibliographical information and synonymy of Magnoliidae. Phytotaxa 19, 71–134. 10.1111/j.1095-8339.2009.00996.x

[B29] Rozas J. Rozas R. (1995). DnaSP, DNA sequence polymorphism: an interactive program for estimating population genetics parameters from DNA sequence data. Comput. Appl. Biosci. 11, 621–625. 10.1093/bioinformatics/11.6.621 8808578

[B30] Scofield S. Dewitte W. Murray J. A. H. (2014). STM sustains stem cell function in the Arabidopsis shoot apical meristem and controls *KNOX* gene expression independently of the transcriptional repressor AS1. Plant Signal. Behav. 9, e28934. 10.4161/psb.28934 24776954 PMC4091562

[B31] Tao S. Khanizadeh S. Zhang H. Zhang S. (2009). Anatomy, ultrastructure and lignin distribution of stone cells in two Pyrus species. Plant Sci. 176, 413–419. 10.1016/j.plantsci.2008.12.011

[B32] Testone G. Condello E. Verde I. Nicolodi C. Caboni E. Dettori M. T. (2012). The peach (*Prunus persica* L. Batsch) genome harbours 10 *KNOX* genes, which are differentially expressed in stem development, and the class 1 *KNOPE1* regulates elongation and lignification during primary growth. J. Exp. Bot. 63, 5417–5435. 10.1093/jxb/ers194 22888130 PMC3444263

[B33] Tian L. Dong X. Cao Y. Zhang Y. Qi D. (2017). Correlation of flesh in pyrus fruit with its stone cells lignin. Southwest China Journal of Agricultural Sciences 30, 2091–2096. 10.16213/j.cnki.scjas.2017.9.028

[B34] Townsley B. T. Sinha N. R. Kang J. (2013). *KNOX1* genes regulate lignin deposition and composition in monocots and dicots. Front. Plant Sci. 4, 1–11. 10.3389/fpls.2013.00121 23653631 PMC3642508

[B35] Wu J. Wang Z. Shi Z. Zhang S. Ming R. Zhu S. (2013). The genome of the pear (Pyrus bretschneideri Rehd.). Genome Res. 23, 396–408. 10.1101/gr.144311.112 23149293 PMC3561880

[B36] Wuddineh W. A. Mazarei M. Zhang J. Y. Turner G. B. Sykes R. W. Decker S. R. (2016). Identification and overexpression of a Knotted1-like transcription factor in switchgrass (*Panicum virgatum* L.) for lignocellulosic feedstock improvement. Front. Plant Sci. 7, 1–15. 10.3389/fpls.2016.00520 27200006 PMC4848298

[B37] Xue C. Yao J.-L. Qin M.-F. Zhang M.-Y. Allan A. C. Wang D.-F. (2018). *PbrmiR397a* regulates lignification during stone cell development in pear fruit. Plant Biotechnol. J. 17, 103–117. 10.1111/pbi.12950 29754465 PMC6330545

[B38] Yan C. Yin M. Zhang N. Jin Q. Fang Z. Lin Y. (2014). Stone cell distribution and lignin structure in various pear varieties. Sci. Hortic. (Amsterdam). 174, 142–150. 10.1016/j.scienta.2014.05.018

[B39] Yoon J. Cho L.-H. Antt H. W. Koh H.-J. An G. (2017). KNOX Protein OSH15 induces grain shattering by repressing lignin biosynthesis genes. Plant Physiol. 174, 312–325. 10.1104/pp.17.00298 28351912 PMC5411160

[B40] Zhang W. Yan H. Chen W. Liu J. Jiang C. Jiang H. (2014). Genome-wide identification and characterization of maize expansin genes expressed in endosperm. Mol. Genet. Genomics 289, 1061–1074. 10.1007/s00438-014-0867-8 25213600

[B41] Zhang J. Cheng X. Jin Q. Su X. Li M. Yan C. (2017). Comparison of the transcriptomic analysis between two Chinese white pear (*Pyrus bretschneideri* Rehd.) genotypes of different stone cells contents. PLoS One 12, 1–22. 10.1371/journal.pone.0187114 PMC5663431 29088238

[B42] Zhao Q. Dixon R. A. (2011). Transcriptional networks for lignin biosynthesis: more complex than we thought? Trends Plant Sci. 16, 227–233. 10.1016/j.tplants.2010.12.005 21227733

[B43] Zhao S. G. Zhang J. G. Zhao Y. P. Zhang Y. X. (2013). New discoveries of stone cell differentiation in fruitlets of “Yali” pears (*Pyrus bretschneideri* Rehd.). J. Food, Agric. Environ. 11, 937–942.

