# Supplementary material for: In Silico Genome-Wide Analysis of the Pear (Pyrus bretschneideri) KNOX Family and the Functional Characterization of PbKNOX1, an Arabidopsis BREVIPEDICELLUS Orthologue Gene, Involved in Cell Wall and Lignin Biosynthesis
Source: Front Genet. 2019 Jul 5;10:632. doi: 10.3389/fgene.2019.00632 (PMC6624237; doi:10.3389/fgene.2019.00632)
Supplement: Supplementary Figure 1 — The exon–intron structure and conserved motifs of PbKNOXs based on the evolutionary relationship. (A) Phylogenetic tree of PbKNOX family members. (B) Conserved motifs of PbKNOX family members. (C) The exon-intron structure of PbKNOX family members. [file Table_1.docx]

***Supplementary Material***

***In silico* genome-wide analysis of the pear (*Pyrus bretschneideri*) *KNOX* family and the functional characterization of *PbKNOX1*, an Arabidopsis *BREVIPEDICELLUS* orthologue gene, involved in cell wall and lignin biosynthesis**

Xi Cheng^1#^, Manli Li^1#^, Muhammad Abdullah^1^, Guohui Li^1^, Jingyun Zhang^1,2^, Muhammad Aamir Manzoor^1^, Han Wang^1^, Qing Jin^1^, Taoshan Jiang^1^, Yongping Cai^1*^, Dahui Li^1*^, Yi Lin^1^

^1^ School of Life Science, Anhui Agricultural University, No. 130, Changjiang West Road, Hefei 230036, China;

^2^ Horticultural Institute, Anhui Academy of Agricultural Sciences, Hefei, Anhui 230031, China;

^#^ These authors contributed equally to this work.

^*^ Corresponding author:

Pro. Yongping Cai

ypcaiah@163.com (Y.C.)

Pro. Dahui Li

dahui2@126.com (D.L.)


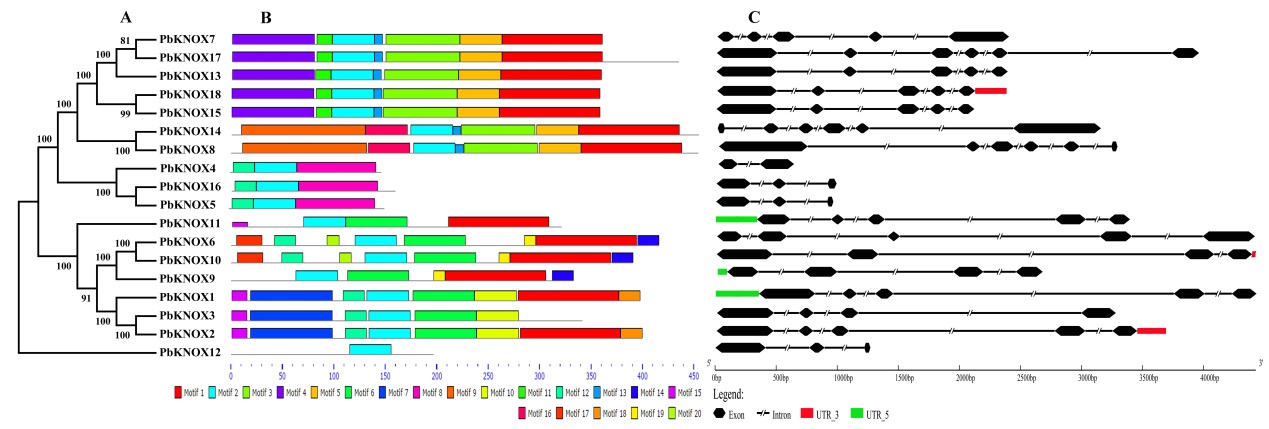
**Supplementary Figures**

**Supplementary Figure 1 The exon-intron structure and conserved motifs of *PbKNOX*s based on the evolutionary relationship.** (A) Phylogenetic tree of *PbKNOX* family members. (B) Conserved motifs of *PbKNOX* family members. (C) The exon-intron structure of *PbKNOX* family members.

**
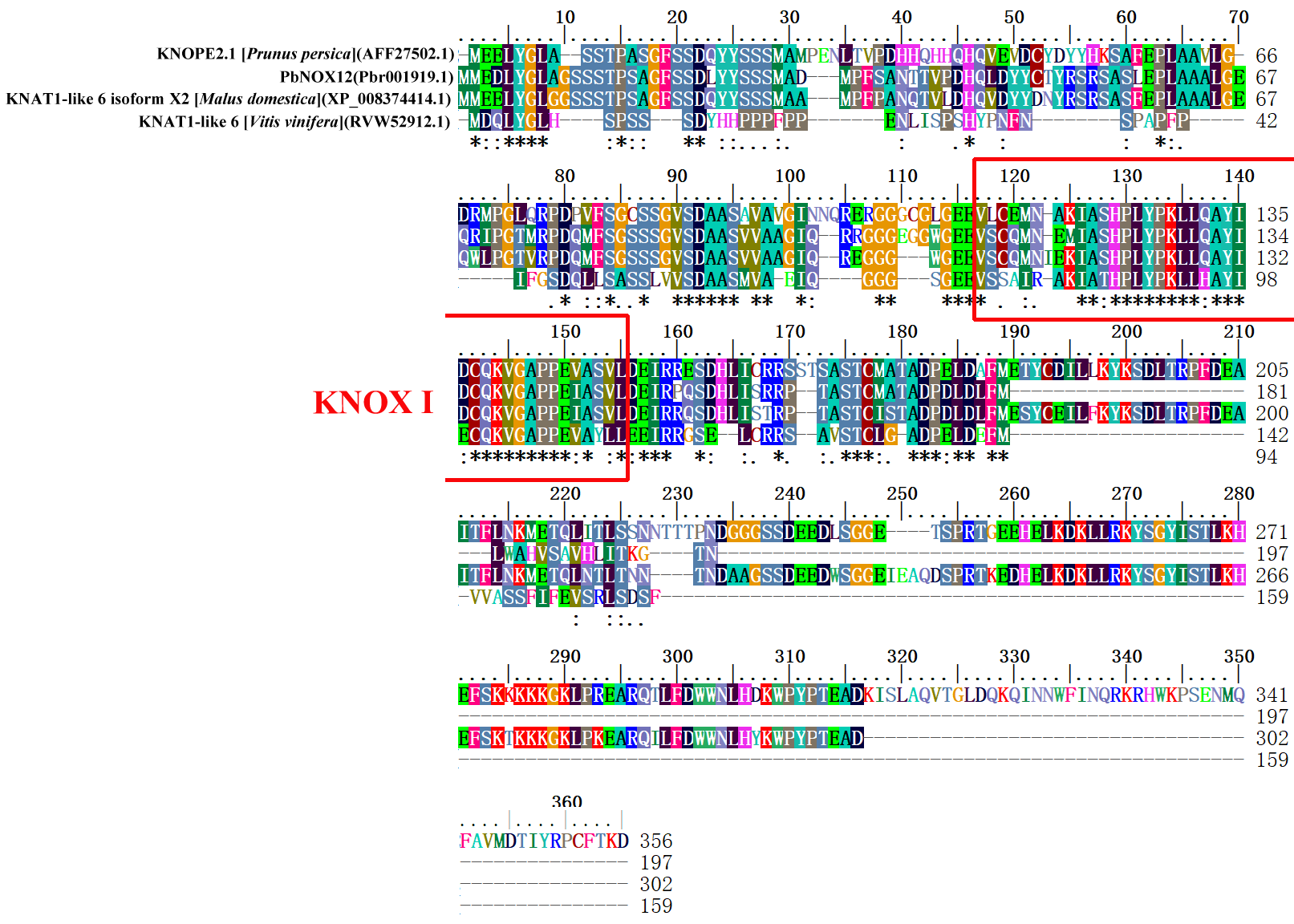
**

**Supplementary Figure 2 Multiple sequence alignment of PbKNOX12 with its orthologs.**


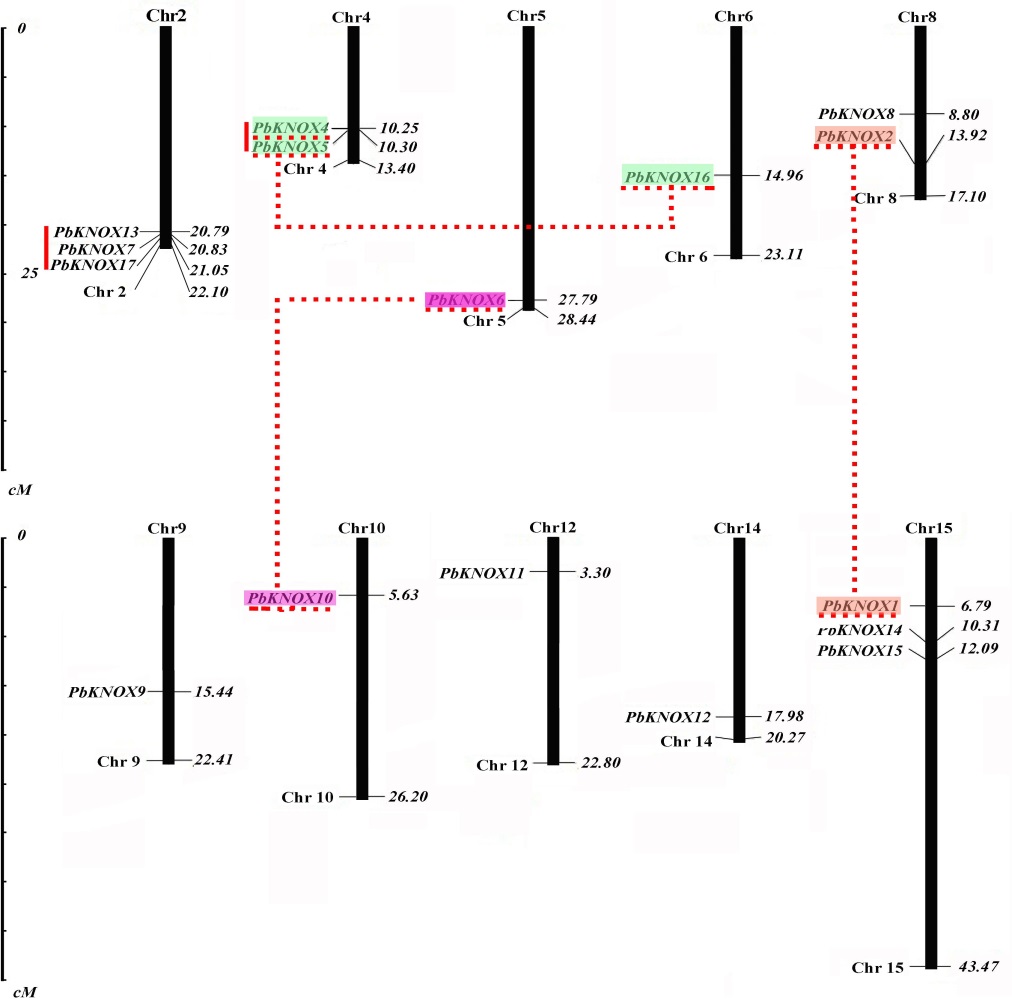


**Supplementary Figure 3 Chromosomal locations and gene duplications of the *PbKNOX*s on the ten chromosomes.** Genes involved in segmental duplication are joined by dashed lines, and the red lines indicate gene clusters. The number corresponding to each gene name represents its position along the chromosome, and the number corresponding to each chromosome name represents the total length of the chromosome.

**
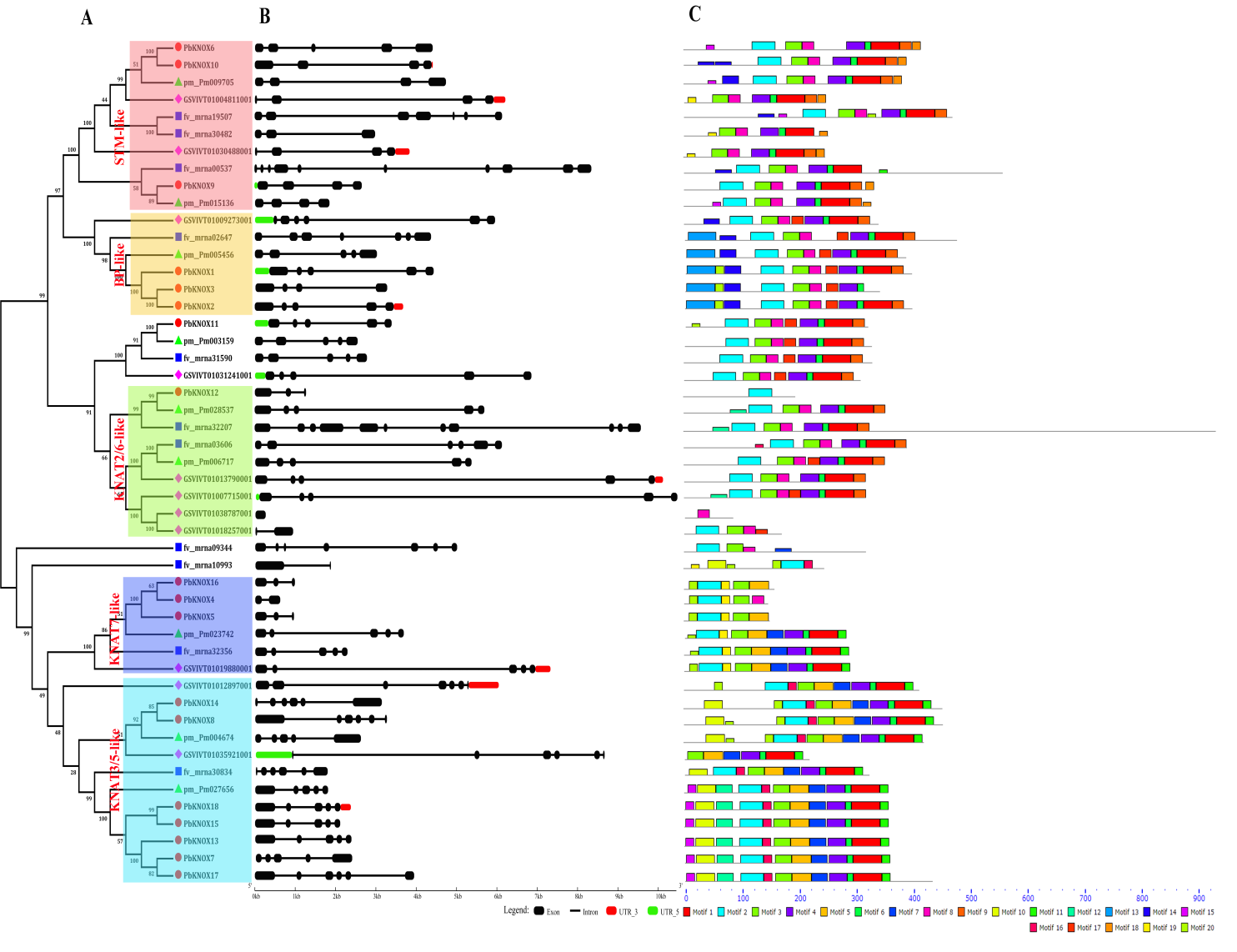
Supplementary Figure 4 Interspecies phylogenetic tree, gene structures and conserved motifs of *KNOX* family members in four rosids.** (A) Phylogenetic tree of *KNOX* family members in four rosids. (B) The exon-intron structure of *KNOX* family members. (C) Conserved motifs of *KNOX* family members.

**
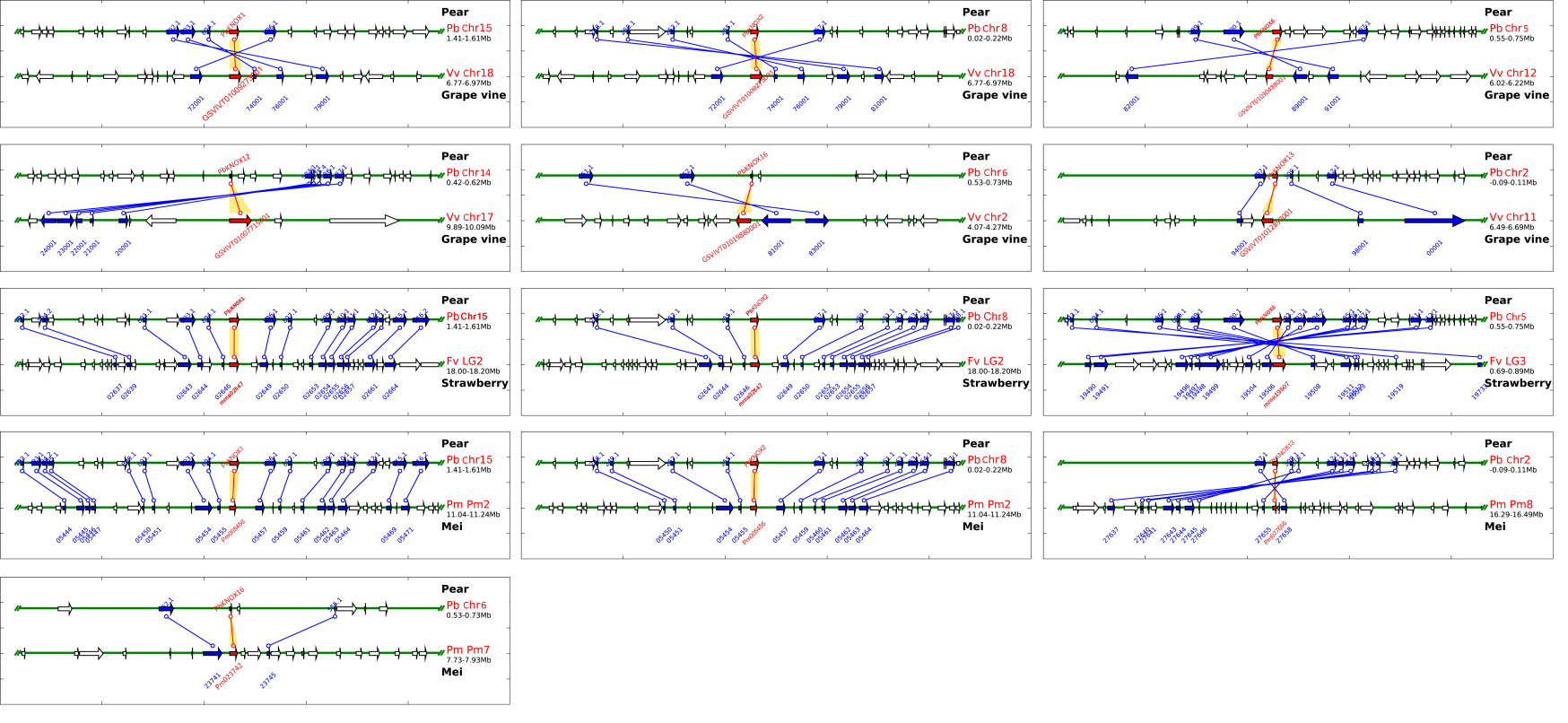
Supplementary Figure 5 Microsynteny regions of *KNOX*s among *Pyrus bretschneideri*, *Fragaria vesca*, *Prunus mume* and *Vitis vinifera*.** The green bars represent chromosomes, and there are chromosomes types and regions on the right. The numbers on both sides of the chromosome are the suffixes of each gene Genome ID. Homologous genes pairs is connected by a straight line, except that *KNOX* is red, and other homologous genes is expressed in blue, and non-homologous genes is expressed in white.

**
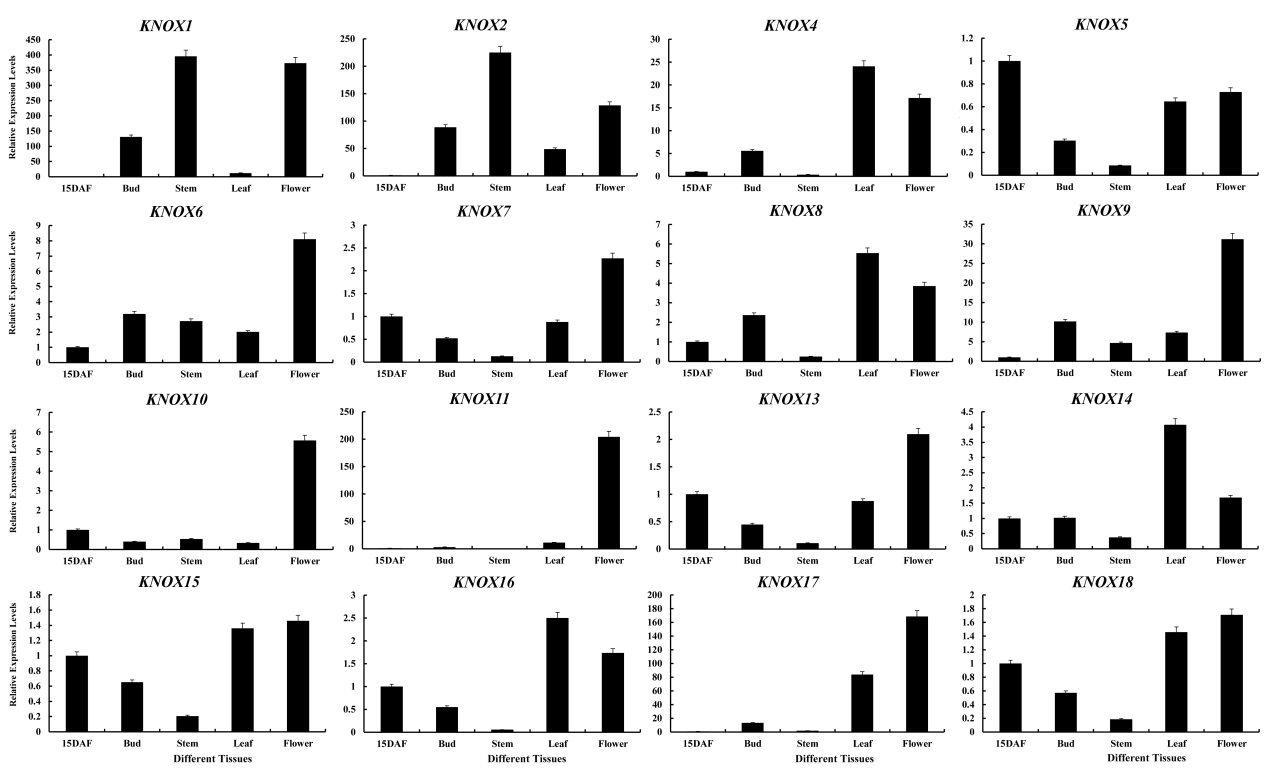
**

**Supplementary Figure 6 Expression patterns of *PbKNOX*s in pear in different tissues.** In this study, *Tubulin* (accession No. AB239680.1) was used as an internal reference. Each qRT-PCR analysis was performed in triplicate. 15 DAF: 15 DAF pear fruits.


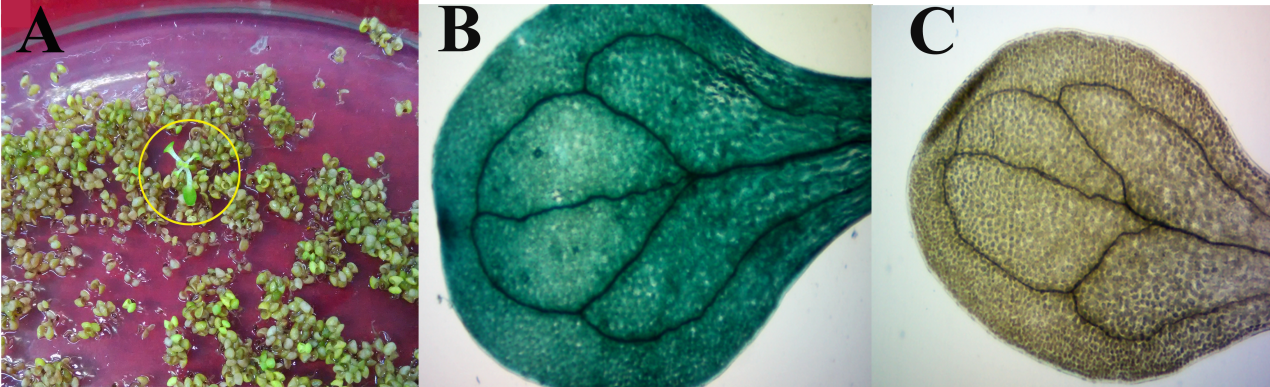


**D**

**
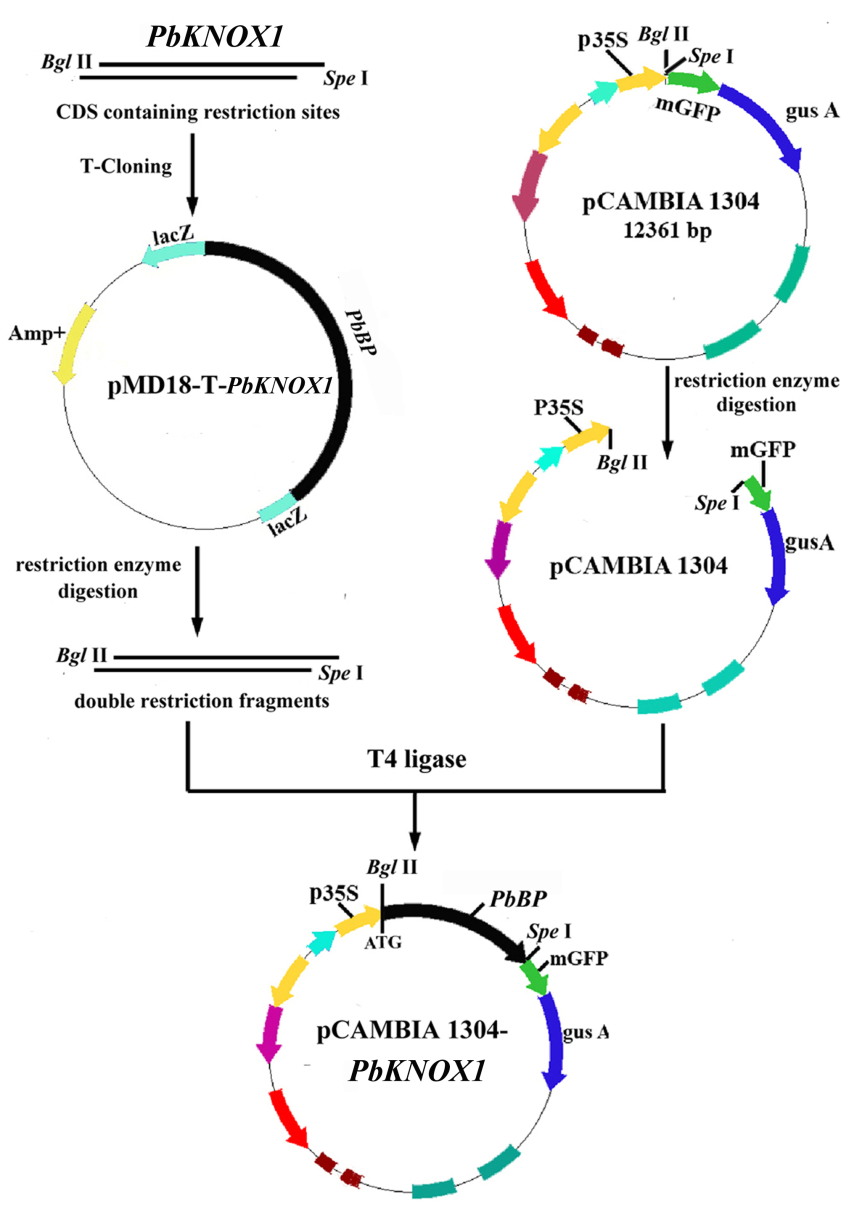
**

**Supplementary Figure 7 Acquisition and confirmation of transgenic Arabidopsis seedlings.** A: Obtaining hygromycin-resistant Arabidopsis seedlings; B: The GUS staining of transgenic Arabidopsis seedling; C: The GUS staining of wild-type Arabidopsis seedling; D: Construction of the eukaryotic expression vector pCAMBIA1304-*PbKNOX1*.

**
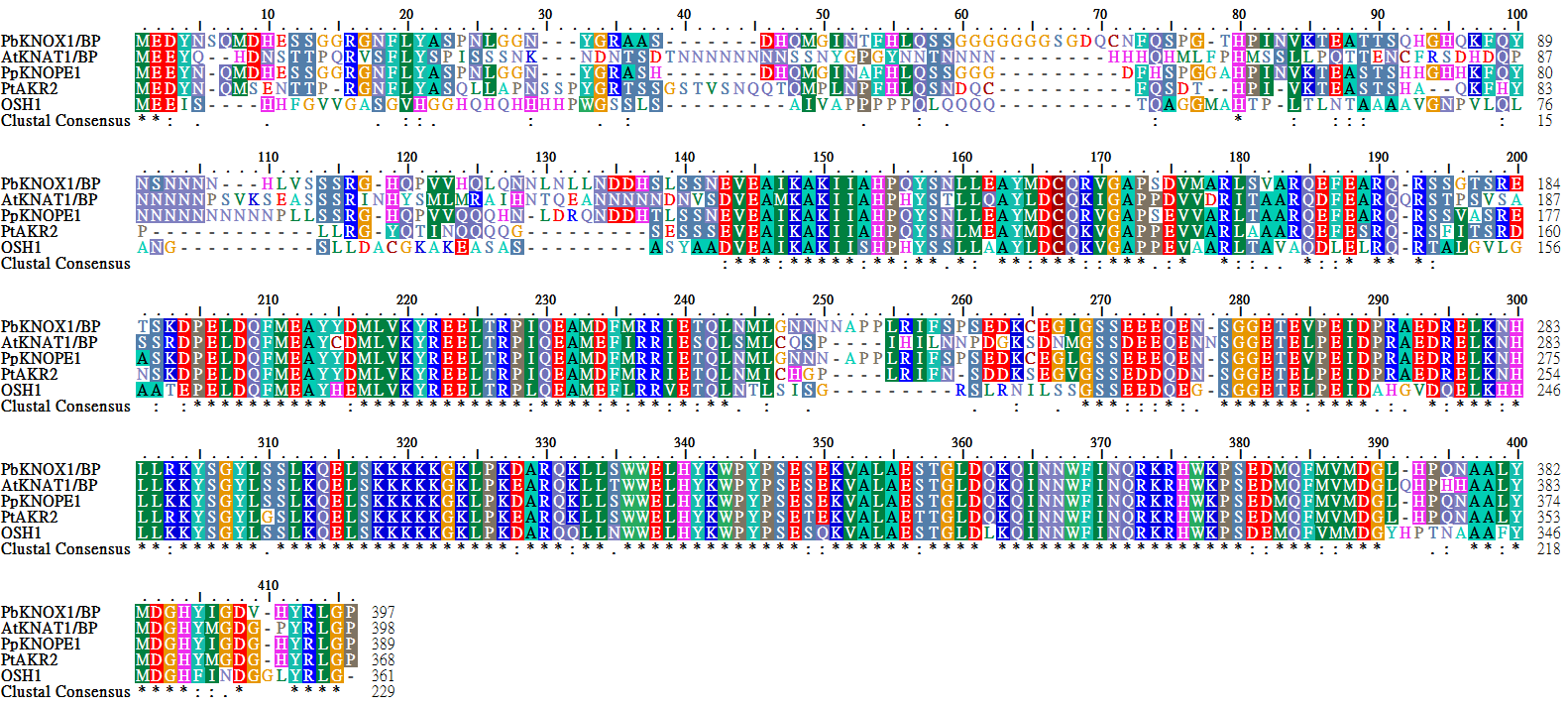
**

**Supplementary Figure 8 Sequence alignment of PbKNOX/BP and other lignin/cell wall biosynthesis-related KNOXs.**

**
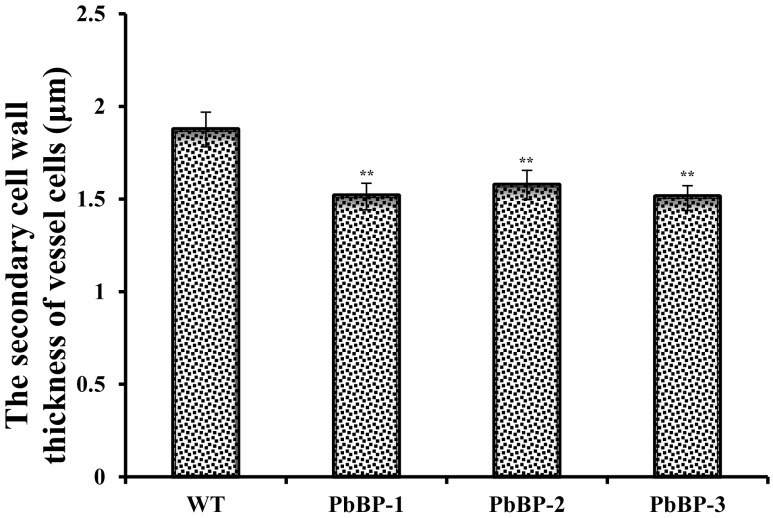
**

**Supplementary Figure 9 Statistical analysis of the secondary cell wall thickness of vessel cells in WT and *PbKNOX1/BP*-overexpressing transgenic plants.** ** Significant difference between the secondary cell wall thickness of the WT and transgenic plants (*P* < 0.01). WT: Wild-type Arabidopsis; PbBP-1~3: *PbKNOX1/BP*-overexpressing transgenic lines.
