# Supplementary material for: In Silico Genome-Wide Analysis of the Pear (Pyrus bretschneideri) KNOX Family and the Functional Characterization of PbKNOX1, an Arabidopsis BREVIPEDICELLUS Orthologue Gene, Involved in Cell Wall and Lignin Biosynthesis
Source: Front Genet. 2019 Jul 5;10:632. doi: 10.3389/fgene.2019.00632 (PMC6624237; doi:10.3389/fgene.2019.00632)
Supplement: Supplementary Table 1 — GenBank accession codes used for constructing phylogenetic trees. [file Table_2.docx]

***Supplementary Material***

***In silico* genome-wide analysis of the pear (*Pyrus bretschneideri*) *KNOX* family and the functional characterization of *PbKNOX1*, an Arabidopsis *BREVIPEDICELLUS* orthologue gene, involved in cell wall and lignin biosynthesis**

Xi Cheng^1#^, Manli Li^1#^, Muhammad Abdullah^1^, Guohui Li^1^, Jingyun Zhang^1,2^, Muhammad Aamir Manzoor^1^, Han Wang^1^, Qing Jin^1^, Taoshan Jiang^1^, Yongping Cai^1*^, Dahui Li^1*^, Yi Lin^1^

^1^ School of Life Science, Anhui Agricultural University, No. 130, Changjiang West Road, Hefei 230036, China;

^2^ Horticultural Institute, Anhui Academy of Agricultural Sciences, Hefei, Anhui 230031, China;

^#^ These authors contributed equally to this work.

^*^ Corresponding author:

Pro. Yongping Cai

ypcaiah@163.com (Y.C.)

Pro. Dahui Li

dahui2@126.com (D.L.)

**Supplementary Tables**

**Supplementary Table 1 GenBank accession codes used for constructing phylogenetic trees.**

| **Gene name** | **Accession number** | **Species name** |
| --- | --- | --- |
| *AtBP* | At4g08150 | *Arabidopsis thaliana* |
| *AtKNAT2* | At1g70510 | *Arabidopsis thaliana* |
| *AtKNAT3* | At5g25220 | *Arabidopsis thaliana* |
| *AtKNAT4* | At5g11060 | *Arabidopsis thaliana* |
| *AtKNAT5* | At4g32040 | *Arabidopsis thaliana* |
| *AtKNAT6* | At1g23380 | *Arabidopsis thaliana* |
| *AtKNAT7* | At1g62990 | *Arabidopsis thaliana* |
| *AtSTM* | At1g62360 | *Arabidopsis thaliana* |
| *AtKNATM* | At1g146760 | *Arabidopsis thaliana* |
| *ARK1* | AY755413 | *Populus* |
| *ARK2* | Potri.002G113300.1 | *Populus* |
| *OSH1* | Os03g51690 | *Oryza sativa* |
| *OSH10* | Os03g47016 | *Oryza sativa* |
| *OSH15* | Os07g03770 | *Oryza sativa* |
| *OSH3* | Os03g51710 | *Oryza sativa* |
| *HOS58* | LOC_Os02g08544.1 | *Oryza sativa* |
| *HOS59* | LOC_Os06g43860.1 | *Oryza sativa* |
| *HOS66* | LOC_Os03g03164.2 | *Oryza sativa* |
| *LePTS* | ACA61779 | *Solanum lycopersicum* |
| *LeT6* | AAC49917 | *Solanum lycopersicum* |
| *LeT12* | AAC49918 | *Solanum lycopersicum* |
| *LeTKn1* | AAC49251 | *Solanum lycopersicum* |
| *LetKn2* | AAD00251 | *Solanum lycopersicum* |
| *LeTKn3* | AAD00252 | *Solanum lycopersicum* |
| *LeTKn4* | AAO33774 | *Solanum lycopersicum* |
| *MtKNOX1* | ABO33478 | *Medicago truncatula* |
| *MtKNOX4* | ABO33481 | *Medicago truncatula* |
| *MtKNOX5* | ABO33482 | *Medicago truncatula* |
| *MtKNOX6* | ABO33483 | *Medicago truncatula* |
| *NtH1* | AAO11694 | *Nitotiana tabacum* |
| *NtH9* | BAA76903 | *Nitotiana tabacum* |
| *NtH15* | BAA25546 | *Nitotiana tabacum* |
| *NtH20* | BAA76904 | *Nitotiana tabacum* |
| *NtH201* | BAF95776 | *Nitotiana tabacum* |
| *NtH22* | BAA76905 | *Nitotiana tabacum* |
| *NtH23* | BAA25921 | *Nitotiana tabacum* |
| *NtKn1* | AF544052 | *Nitotiana tabacum* |
| *PpKNOPE1* | ABD52723 | *Prunus persica* |
| *PpKNOPE2* | ABO28750 | *Prunus persica* |
| *PpKNOPE2.1* | JQ038131 | *Prunus persica* |
| *PpKNOPE3* | ACJ71731 | *Prunus persica* |
| *PpKNOPE4* | ABO26062 | *Prunus persica* |
| *PpKNOPE6* | ADC35598 | *Prunus persica* |
| *PpKNOPE7* | JQ038132 | *Prunus persica* |
| *PpKNOPEM* | JQ038133 | *Prunus persica* |
| *PpSTMlike1* | ADC35599 | *Prunus persica* |
| *PpSTMlike2* | ADC35600 | *Prunus persica* |
| *ZmRS1* | NP_001149651 | *Zea mays* |
| *Zmkn1* | NP_001105436 | *Zea mays* |
| *GmKNOX1* | Glyma09g01000.1 | *Glycine max* |
| *GmKNOX2* | Glyma15g11850.1 | *Glycine max* |
| *GmKNOX3* | Glyma07g39350.1 | *Glycine max* |
| *GmKNOX4* | Glyma17g01370.1 | *Glycine max* |
| *GmKNOX5* | Glyma14g05150.1 | *Glycine max* |
| *GmKNOX6* | Glyma14g10430.1 | *Glycine max* |
| *GmKNOX7* | Glyma04g05210.1 | *Glycine max* |
| *GmKNOX8* | Glyma08g39170.1 | *Glycine max* |
| *GmKNOX9* | Glyma02g04190.1 | *Glycine max* |
| *GmKNOX10* | Glyma01g03450.1 | *Glycine max* |
| *GmKNOX11* | Glyma19g41610.1 | *Glycine max* |
| *GmKNOX12* | Glyma10g28820.1 | *Glycine max* |
| *GmKNOX14* | Glyma04g35850.1 | *Glycine max* |
| *GmKNOX16* | Glyma04g06810.1 | *Glycine max* |
| *GmKNOX17* | Glyma17g32980.1 | *Glycine max* |
| *GmKNOX18* | Glyma14g13750.1 | *Glycine max* |
| *GmKNOX19* | Glyma09g12820.1 | *Glycine max* |
| *GmKNOX20* | Glyma13g22530.1 | *Glycine max* |
| *GmKNOX21* | Glyma17g11330.1 | *Glycine max* |
| *GmKNOX23* | Glyma05g03650.1 | *Glycine max* |
| *GmKNOX24* | Glyma17g14180.1 | *Glycine max* |
| *GmKNOX25* | Glyma01g42410.1 | *Glycine max* |
| *GmKNOX26* | Glyma11g02960.1 | *Glycine max* |
| *GmKNOX27* | Glyma0041s00360.1 | *Glycine max* |
| *GhKNL1* | KC200250 | *Gossypium hirsutum* L. |

**Supplementary Table 2 Primers for qRT-PCR and vector construction.**

| **Primer name** | **Primer sequence (5*'*~3*'*)** |
| --- | --- |
| *PbKNOX1-*F  *PbKNOX1-*R | TCATAAATCAAAGGAAGAGGCACTGGAA  CAATGTCACGGAGTTGTTTAGGTCA |
| *PbKNOX2*-F | ATGGAAGGTGTTGGTGCCAAT |
| *PbKNOX2*-R | TCAGAGCAATGTGGGAGAGATATCC |
| *PbKNOX3*-F | GCCATATCCTTCGGTATA |
| *PbKNOX3*-R | TCCTCTATGATCCAAGAAC |
| *PbKNOX4*-F  *PbKNOX4*-R | AGGCTCTTACTGGTATGC  AATAGTCGAGATTCAGAGTCT |
| *PbKNOX5*-F  *PbKNOX5*-R | AGGCTCTTACTGGCATAA  ACCCACTTCTTACAAGGA |
| *PbKNOX6* -F | CGGTCACTACTACATGGA |
| *PbKNOX6*-R | CATACTAATTGCTTGTCATCTT |
| *PbKNOX7*-F | GAATTGGCACACCAACATT |
| *PbKNOX7*-R | TTATGTTAATCGTAATCCTACCTC |
| *PbKNOX8*-F | CACTCTGATTCTGATGTTG |
| *PbKNOX8*-R | ATACGCTTCGATCTTCTG |
| *PbKNOX9*-F | ATCAACACCATCACTATC |
| *PbKNOX9*-R | ATACAGAAGCCCTTTATC |
| *PbKNOX10*-F | CCACTATTACATGGACAGT |
| *PbKNOX10*-R | GGCATTCCTCAATAACAC |
| *PbKNOX11*-F | ATCGGAGGACCTATGATG |
| *PbKNOX11*-R | CAACCAAATACTTGATACAATGA |
| *PbKNOX12*-F | TCAGAGCAATGTGGGAGAGATATCC |
| *PbKNOX12*-R | ATGGAAGGTGTTGGTGCCAAT |
| *PbKNOX13*-F | GAATTGGCACACCAACATT |
| *PbKNOX13*-R | TTATGTTAATCGTAATCCTACCTC |
| *PbKNOX14*-F | AATCAGAGGAAGAGGAACT |
| *PbKNOX14*-R | TACTATAACGGCTCATATTGG |
| *PbKNOX15*-F | ACAGCAACATTTCATCAACTT |
| *PbKNOX15*-R | GCCAATCAATGCGAACAA |
| *PbKNOX16*-F | GAGTGCTGTCCTTGTAAG |
| *PbKNOX16*-R | AACACTACACTTTGATTGATTAG |
| *PbKNOX17*-F | TTCGTGACCATTGTTGTT |
| *PbKNOX17*-R | ACTTCTTCTACGTCTTCTTG |
| *PbKNOX18*-F | GAATTGGCACAGCAACATT |
| *PbKNOX18*-R | AACAACCATACATAATCCTACCT |
| *Tubulin*-F | AGAACAAGAACTCGTCCTAC |
| *Tubulin*-R | GAACTGCTCGCTCACTCTCC |
| *PbKNOX1*-full length-F | ATGGAAGACTACAACAGTCAAATGGATCATG |
| *PbKNOX1*-full length-R | TCATGGCCCGAGACGGTAGTGAACG |
| *PbKNOX1*-ZH-F | GAAGATCTGATGGAAGACTACAACAGTCAAATGGATCATG |
| *PbKNOX1*-ZH-R | GGACTAGTTGGCCCGAGACGGTAGTGAACG |
| *β-AtTubulin4-F*  *β-AtTubulin4-R* | GGAGCTACGCAGAACAACTAAGA  CCCACGAGGATCATAGTTGCAACTGA |
| *AtPAL1- F* | AAGATTGGAGCTTTCGAGGA |
| *AtPAL1-R* | TCTGTTCCAAGCTCTTCCCT |
| *AtC4H-F*  *AtC4H-R*  *At4CL1-F*  *At4CL1-R*  *AtHCT-F*  *AtHCT-R* | ACTGGCTTCAAGTCGGAGAT  ACACGACGTTTCTCGTTCTG  TCAACCCGGTGAGATTTGTA  TCGTCATCGATCAATCCAAT  GCCTGCACCAAGTATGAAGA  GACAGTGTTCCCATCCTCCT |
| *AtC3H1-F* | GTTGGACTTGACCGGATCTT |
| *AtC3H1-R* | ATTAGAGGCGTTGGAGGATG |
| *AtCCOMT1-F* | CTCAGGGAAGTGACAGCAAA |
| *AtCCOMT1-R* | GTGGCGAGAAGAGAGTAGCC |
| *AtCCR1-F* | GTGCAAAGCAGATCTTCAGG |
| *AtCCR1-R* | GCCGCAGCATTAATTACAAA |
| *AtF5H-F* | CTTCAACGTAGCGGATTTCA |
| *AtF5H-R* | AGATCATTACGGGCCTTCAC |
| *AtCOMT1-F* | TTCCATTGCTGCTCTTTGTC |
| *AtCOMT1-R* | CATGGTGATTGTGGAATGGT |
| *AtCAD5-F* | TTGGCTGATTCGTTGGATTA |
| *AtCAD5-R* | ATCACTTTCCTCCCAAGCAT |
| *AtCAD4-F* | CACTTCGGTCTAATGGCGAGT |
| *AtCAD4-R* | AGTCAAGGGAATCTGCGAGTCT |

**Supplementary Table 3 Details of 20 conserved motifs in the PbKNOXs.**

| **Motif** | **Width** | **Best Possible Match** | **Domain** |
| --- | --- | --- | --- |
| 1 | 98 | ELKHELKQKYKEYIVDIREEIMRKRRAGKLPKDTTSVLKSWWQSHYKWPYPTEEDKARLVQETGLQLKQINNWFINQRKRNWHPNIDMTFVLKDKRKR | ELK, Homeobox_KN (HOMEODOMAIN) |
| 2 | 41 | FKAKIIAHPLYEQLVSAYVSCQRVATPVDQLPRIDEQCVQS | KNOX1 |
| 3 | 72 | ANGDVGVMDEKELDLFMTNYVLLLCSFKEQLQQHVRVHAMEAVMACWELDQSLQSLTGVSTGEGTGATMSDD | KNOX2 |
| 4 | 80 | MAFHHHHQQTPHEMAFHSFAEDQPPLSGAPTWLNNAAFRQQNTSFGGLHDAGRNDDVVISPSGKSSNCSDRNRRDISGYD | * |
| 5 | 41 | DDQVDSDINSYDGSLDGPDTMGFGPLVPTESERSLMERVRQ | * |
| 6 | 60 | RSSGTSGETGKDPELDQFMEAYCEMLTKYREELTKPFKEAMIFMRRIETQLKMLTNNNNA | KNOX2 |
| 7 | 80 | FLYASPNLGGNYGRAASDHQMGINTFHLQSSGGGGGGGSGDQCNFQSPGAHPINVKTEATTSQHGHPKFQYNNNNNNHHH | Low complexity region |
| 8 | 77 | HHLLRSYASQQQHGQSVSPHERQELDNFLAQYLIVLCSFKEHLQQHVRVHAVEAVMACREIESNLQALTGIPIRLFW | KNOX2 |
| 9 | 121 | PLHHFTDQTQQQHQQYQSDQPDPNSKPPEPHHPFQPAPNWLNSALLRNFTNTDTNPTNTNNANNNGGGVSNFLNLHVTASDSVASQASNQWLSQSHRPILHRNHSDVIDDVTVAGDSMIAA | Low complexity region |
| 10 | 41 | PPLRIFSPSEDKCEGIGSSEDEQENSGGETEVPEIDPRAED | * |
| 11 | 15 | EEEEGEDELECESAR | * |
| 12 | 21 | HQLQMNLNPGNDGHHTSTNRQ | * |
| 13 | 8 | QRVVDKYS | * |
| 14 | 21 | HYYMDSVMGNPFPMDISPTLL | * |
| 15 | 15 | MEDYNSQMDHESSGG | * |
| 16 | 41 | MSHDSADLKPDTNLNKNDGGVVESGIPGGGGGGDGGVINWQ | * |
| 17 | 25 | NGTCSMMAFGENSSNGGGMCMMMMM | * |
| 18 | 21 | QNAALYMDGHYIGDGHYRLGP | * |
| 19 | 11 | NNFIDPQAEDR | * |
| 20 | 12 | AAASYFMDNNNN | * |

Note: ‘*’ indicates that the motif has no specific annotation information.

**Supplementary Table 4 Ka/Ks analysis for *KNOX* duplicated genes of pear.**

| **Duplicated gene pairs** | | **Ka** | **Ks** | **Ka/Ks** | **Purifying selection** | **Duplicated type** |
| --- | --- | --- | --- | --- | --- | --- |
| ***PbKNOX5*** | ***PbKNOX16*** | 0.0180 | 0.1383 | 0.1367 | **Yes** | **Segmental** |
| ***PbKNOX6*** | ***PbKNOX10*** | 0.0307 | 0.2081 | 0.1475 | **Yes** | **Segmental** |
| ***PbKNOX8*** | ***PbKNOX14*** | 0.0422 | 0.2545 | 0.1658 | **Yes** | **Segmental** |

**Supplementary Table 6 Collinearities were identified in four rosids.**

| **collinear gene pairs (pear/strawberry)** | | **collinear gene pairs**  **(pear/mei)** | | **collinear gene pairs**  **(pear/grape)** | |
| --- | --- | --- | --- | --- | --- |
| PbKNOX1 | mrna02647 | PbKNOX1 | Pm005456 | PbKNOX1 | GSVIVT01009273001 |
| PbKNOX2 | mrna02647 | PbKNOX2 | Pm005456 | PbKNOX2 | GSVIVT01009273001 |
| PbKNOX6 | mrna19507 | PbKNOX3 | Pm027656 | PbKNOX6 | GSVIVT01030488001 |
|  |  | PbKNOX16 | Pm023742 | PbKNOX12 | GSVIVT01007715001 |
|  |  |  |  | PbKNOX13 | GSVIVT01012897001 |
|  |  |  |  | PbrKNOX16 | GSVIVT01019880001 |

**Supplementary Table 7 Putative *cis*-acting regulatory elements in the *PbKNOX* promoters.**

| **Element** | **MBS** | **MRE** | **LTR** | **HSE** | **TC-rich repeat** | **ABRE** | **ERE** | **CGTCA motif** | **TCA-**  **element** |
| --- | --- | --- | --- | --- | --- | --- | --- | --- | --- |
| **Function** | Drought stress | Light response | low-temperature stress | Heat stress | Defense and stress | ABA response | ethylene response | MeJA response | SA response |
| ***PbKNOX1*** | 4 |  | 2 | 3 | 3 | 1 | 1 |  |  |
| ***PbKNOX2*** | 2 |  | 1 | 3 | 4 |  |  | 1 | 2 |
| ***PbKNOX3*** | 3 |  | 1 | 3 | 4 |  |  | 1 | 2 |
| ***PbKNOX4*** | 2 |  |  | 1 | 2 |  |  | 3 | 2 |
| ***PbKNOX5*** | 2 |  |  | 1 | 2 |  |  | 3 | 2 |
| ***PbKNOX6*** | 2 |  | 2 | 6 | 2 | 1 |  |  |  |
| ***PbKNOX7*** | 2 | 1 |  | 2 |  | 7 |  | 1 |  |
| ***PbKNOX8*** | 1 |  |  | 1 | 4 | 1 | 1 | 1 |  |
| ***PbKNOX9*** | 1 |  |  | 4 |  |  | 3 | 1 | 2 |
| ***PbKNOX10*** | 1 |  |  |  | 1 | 1 |  |  | 3 |
| ***PbKNOX11*** | 2 |  |  | 3 | 1 | 6 |  | 3 | 3 |
| ***PbKNOX12*** | 4 | 2 | 2 | 4 |  | 1 |  | 1 | 1 |
| ***PbKNOX13*** | 2 |  |  | 2 |  | 7 |  | 1 |  |
| ***PbKNOX14*** | 4 |  | 2 |  |  | 3 |  | 3 | 1 |
| ***PbKNOX15*** | 1 | 1 | 1 | 4 | 2 | 3 | 3 | 2 |  |
| ***PbKNOX16*** | 1 | 2 |  | 4 | 2 |  |  |  | 2 |
| ***PbKNOX17*** | 2 | 1 |  | 2 |  | 7 |  | 1 |  |
| ***PbKNOX18*** |  | 2 | 1 | 1 | 2 | 6 | 2 | 2 |  |
| **Total** | 36 | 9 | 12 | 44 | 29 | 44 | 10 | 24 | 20 |

**Supplementary Table 8 Sequence identity and similarity among PbKNOXs and *KNOX* protein sequences of various plants.**

| **Iden/Sim（%/%）** | **At**  **BP** | **Pp**  **KNOPE1** | **OSH1** | **ZmKn1** | **PtAKR2** | **Gh**  **KNL1** | **At**  **KNAT7** | **At**  **STM** | **PtAKR1** | **LeT6** |
| --- | --- | --- | --- | --- | --- | --- | --- | --- | --- | --- |
| **PbKNOX1** | **60/91** | **89/94** | 38/68 | 49/82 | **71/85** | 19/58 | 18/28 | 39/48 | 36/75 | 37/74 |
| **PbKNOX2** | **60/70** | **89/95** | 38/68 | 48/81 | **71/84** | 19/58 | 17/28 | 38/48 | 36/77 | 36/75 |
| **PbKNOX3** | 45/55 | 73/80 | 30/49 | 36/60 | 56/67 | 15/44 | 14/24 | 29/37 | 26/50 | 27/48 |
| **PbKNOX4** | 8/12 | 8/30 | 6/30 | 8/32 | 7/29 | 39/48 | 35/38 | 7/12 | 7/31 | 8/33 |
| **PbKNOX5** | 8/12 | 8/25 | 8/31 | 8/33 | 8/30 | 39/48 | 36/40 | 7/12 | 7/31 | 8/33 |
| **PbKNOX6** | 38/47 | 36/60 | 27/53 | 33/59 | 36/59 | 16/47 | 16/28 | 60/68 | 63/74 | 57/69 |
| **PbKNOX7** | 18/32 | 17/50 | 18/57 | 18/59 | 17/55 | 51/78 | 46/56 | 18/31 | 17/56 | 17/63 |
| **PbKNOX8** | 18/31 | 19/52 | 16/56 | 19/64 | 17/62 | 43/61 | 38/46 | 17/29 | 18/61 | 17/63 |
| **PbKNOX9** | 37/45 | 35/59 | 31/56 | 37/64 | 36/61 | 18/50 | 20/31 | 49/58 | 49/66 | 49/68 |
| **PbKNOX10** | 40/49 | 38/63 | 29/66 | 36/77 | 39/74 | 17/58 | 16/28 | 58/60 | 68/84 | 59/84 |
| **PbKNOX11** | 29/42 | 32/60 | 33/70 | 31/72 | 32/68 | 23/70 | 23/35 | 33/43 | 31/68 | 34/73 |
| **PbKNOX12** | 5/10 | 5/33 | 6/40 | 7/37 | 6/35 | 6/49 | 7/16 | 6/12 | 7/38 | 5/37 |
| **PbKNOX13** | 18/32 | 17/49 | 18/51 | 18/52 | 17/49 | 51/72 | 46/56 | 18/31 | 17/48 | 17/52 |
| **PbKNOX14** | 18/31 | 18/52 | 16/57 | 18/65 | 17/62 | 42/61 | 39/46 | 17/29 | 17/61 | 16/63 |
| **PbKNOX15** | 18/31 | 17/50 | 17/57 | 19/60 | 16/56 | 52/78 | 46/56 | 18/31 | 17/57 | 17/62 |
| **PbKNOX16** | 8/12 | 8/26 | 8/28 | 8/28 | 7/27 | 39/48 | 35/40 | 7/13 | 7/26 | 8/29 |
| **PbKNOX17** | 16/29 | 15/57 | 16/55 | 16/56 | 15/55 | 43/65 | 38/46 | 17/29 | 15/54 | 15/60 |
| **PbKNOX18** | 18/31 | 17/50 | 18/57 | 19/60 | 71/84 | 19/58 | 46/56 | 18/31 | 17/57 | 17/62 |

**Supplementary Table 9 Potential KNOX binding sites in pear lignin genes.**

| **Pear lignin genes** | **Potential KNOX binding sites** | |
| --- | --- | --- |
|  | **Gene ID** | **Promoter** |
| *C4H* | Pbr017290.1 | -990 bp |
| *C3H* | Pbr026583.1 | -1823 bp |
| *HCT* | Pbr022422.1 | -1928 bp |
| *CCOMT* | Pbr034039.1 | -925 bp |
| *COMT* | Pbr013510.1 | -700 bp |
| *F5H* | Pbr022142.1 | -1421 bp |
| *CCR1* | Pbr022402.1 | -1849 bp |
| *CCR2* | Pbr022405.1 | -351 bp |
| *CCR3* | Pbr022403.1 | -1668 bp |
| *CAD2* | Pbr026287.1 | -1349 bp |

Note: The location of the binding sites is indicated in bracketed.
